# The effectiveness of exercise snacks as a time-efficient treatment for improving cardiometabolic health in adults: a systematic review and meta-analysis

**DOI:** 10.3389/fcvm.2025.1643153

**Published:** 2025-08-13

**Authors:** Jiping Chen, Yanyu Lu, Haojie Zhao, Haojie Liu, Jiawei Yao

**Affiliations:** ^1^ School of Physical Education, Shandong University, Jinan, China; ^2^ School of Innovation Design, Guangzhou Academy of Fine Arts, Guangzhou, Guangdong, China; ^3^ Culture and Tourism College, Guangdong Vocational Academy of Art, Foshan, Guangdong, China

**Keywords:** physical activity, exercise snacks, cardiometabolic health, time-efficient strategy, adult

## Abstract

**Objectives:**

Exercise snacks (ES) are short bursts of intensive exercise done at regular intervals during the day. The benefits to cardiometabolic health of ES for adults are unclear. The present meta-analysis aimed to assess the effects of ES on cardiometabolic health in adults.

**Methods:**

A systematic search was conducted in accordance with the PRISMA guidelines, using the following databases: PubMed, Web of Science, The Cochrane Library, EBSCOhost, and Embase, spanning all previous years up to May 24, 2025. The included studies were evaluated for their literature quality using the effective public health practice project quality assessment tool. The data were analyzed using a random-effects meta-analysis.

**Results:**

27 studies with a total of 970 participants were included in this meta-analysis. There was a significant beneficial effect of ES on maximal oxygen uptake [SMD (standard mean difference) = 0.63; 95% CI: 0.45, 0.82; *P* < 0.001], body fat percentage (SMD = −0.21; 95% CI: −0.38, −0.04; *P* = 0.02), waist circumference (SMD = −0.21; 95% CI: −0.42, −0.00; *P* = 0.05), systolic blood pressure (SMD = −0.67; 95% CI: −0.98, −0.37; *P* < 0.001), diastolic blood pressure (SMD = −0.34; 95% CI: −0.65, −0.04; *P* = 0.03), fasting blood glucose (SMD = −0.40; 95% CI: −0.79, −0.02; *P* = 0.04), high-density lipoprotein (SMD = 0.26; 95% CI: 0.07, 0.46; *P* = 0.01), low-density lipoprotein (SMD = −0.30; 95% CI: −0.56, −0.04; *P* = 0.02), and total cholesterol (SMD = −0.39; 95% CI: −0.68, −0.10; *P* = 0.01). There are no statistically effects of ES on body mass (SMD = −0.094; 95% CI: −0.261, 0.072; *P* = 0.267) and triglycerides (SMD = −0.193; 95% CI: −0.478 to 0.093; *P* = 0.187). Furthermore, no significant risk of publication bias was observed for any outcomes.

**Conclusion:**

This comprehensive meta-analysis provides robust evidence that ES, characterized by brief, intensive bouts of activity interspersed throughout the day, offer significant and clinically meaningful benefits for improving multiple facets of cardiometabolic health in adults.

## 1 Introduction

Regular physical activity (PA) has been widely studied for its benefits on cardiometabolic health (CMH), including lower risk of hypertension, diabetes, and coronary heart disease (1–3). Unfortunately, approximately one-third (31%) of the global adult population (1.8 billion adults) do not adhere to the World Health Organization (WHO) recommended PA guidelines (150 min/week of moderate-intensity aerobic activity, 75 min/week of vigorous-intensity activity, or equivalent combinations), and this non-adherence rate continues is still growing globally (4, 5). It is essential to note that a lack of time is one of the primary barriers for physically inactive individuals to exercise (6). To this end, several investigators have examined the efficacy of exercise snacks (ES) as an alternative to PA approaches recommended by the WHO (7).

ES refers to the approach of short bouts (≤1 min) of vigorous-intensity (≥64% VO_2_peak, ≥77% of maximal heart rate) PA spread throughout the day (7, 8). In terms of intensity, ES differ from movement breaks, which interrupt prolonged sedentary behavior through light- to moderate-intensity PA (9). In terms of duration, ES also differ from high-intensity interval training (HIIT) or sprint interval training (SIT), which are usually performed in a single session (10). Several research studies have demonstrated that this time efficiency method delivers a number of benefits to inactive adults, including improved body composition (11), cardiorespiratory fitness (12), and endothelial function (7). The accumulation of short-term benefits from short but intense PA can be responsible for these changes (8), whereas the acute improvements in endothelial function have been linked to increased blood-flow-induced shear stress (13). Furthermore, recent research also demonstrates that the benefits of ES appear comparable, or even exceed, to moderate-intensity continuous training (MICT) (14–16). However, ES remains a relatively new, broadly defined and understudied concept (17). The CMH benefits of ES in adults remain unclear.

Therefore, to further examine the potential benefit of ES. We conducted a systematic review and meta-analysis to synthesize the effects of ES on CMH markers, including body mass, waist circumference (WC), body fat percentage (BF%), systolic blood pressure (SBP), diastolic blood pressure (DBP), maximal oxygen uptake (VO_2_max), fasting blood glucose (FBG), total cholesterol (TC), low-density lipoprotein (LDL) cholesterol, high-density lipoprotein (HDL) cholesterol and triglycerides (TG) in adults.

## 2 Methods

### 2.1 Study registration and protocol

This study was conducted in accordance with the Preferred Reporting Items for Systematic Review and Meta-Analysis (PRISMA) guideline (18). The PRISMA 2020 Checklist is available in the Supplementary Table S1. Also, this research has been preregistered at the International Prospective Register of Systematic Reviews (PROSPERO) with the protocol number CRD420251064915.

### 2.2 Information sources and search strategy

A literature search was conducted on March 16, 2024, with an updated search on May 24, 2025, using the following databases: PubMed, Web of Science, The Cochrane Library, EBSCOhost, and Embase. Search terms for each database were identified based on previous systematic reviews. Using Web of Science as an example, we searched for the following terms by title, abstract, and language: TS = (“exercise snack” OR “movement breaks” OR “physical activity breaks” OR “exercise bursts” OR “high-intensity interval training” OR “low-volume high-intensity interval training”) AND TS = (“waist circumference” OR “body weight” OR “fasting blood glucose” OR “total cholesterol” OR “systolic blood pressure” OR “diastolic blood pressure” OR “low-density lipoprotein” OR “high-density lipoprotein” OR “maximal oxygen uptake” OR “triglycerides” OR “body fat percentage”) AND TS = (“clinical trial” OR “randomized controlled trial”) AND LA = (English). The whole search strategy is described in the Supplementary Table S2.

Additionally, the reference lists of the included trials were reviewed to identify potential studies that could also be utilized in the present study. A total of two reviewers (J.C. and J.Y.) were involved in the independent assessment of the title, abstract and full text for eligibility for inclusion. If necessary, a third investigator (H.Z.) was consulted.

### 2.3 Eligibility criteria

To structure the eligibility criteria in the present review, studies needed to meet the following PICOS (Patient/Population; Intervention; Comparison; Outcome; Study design) approach (19): (1) participants: the population includes individuals aged 18 years and older; (2) intervention: intervention takes the form of supervised, high-intensity physical activity lasting ≤1 min throughout the day, which is referred to as exercise snacks; (3) comparison: a control group that maintains their habits without any intervention; (4) outcomes: an assessment of at least one of the following cardiometabolic health markers in humans: VO_2_max, body mass, BF%, WC, SBP, DBP, FBG, HDL, LDL, TC, TG; (5) study design: the study included a quantitative analysis of the effect of ES on at least one of the following CMH outcome measures: VO_2_max, body mass, BF%, WC, SBP, DBP, FBG, HDL, LDL, TC, TG.

### 2.4 Data collection process

Data for each research were independently retrieved by two assessors (H.L. and Y.L.), and the following information was gathered: (1) surname of the primary author; (2) year of publication; (3) country of the study; (4) type of study design; (5) number of participants, including gender, age, and disease status in both intervention and control groups; (6) characteristics of interventions concerning mode, frequency, duration, intensity, and content; (7) cardiometabolic health-related outcomes; (8) mean, standard deviation, and sample size recorded for each group prior to and following the intervention. The data were used to calculate the differences in each outcome indicator before and after the intervention. Calculations were executed as detailed below.

First, the formula for calculating the difference in means is as follows:

Mdiff=Mpost−Mpre

Mdiff represents the raw mean difference, Mpost denotes the reported mean post-intervention, and Mpre signifies the reported mean pre-intervention (20).

Then the SD of the difference in means (SDdiff) is computed as follows:

$$\mathrm{SDdiff}=\sqrt{\mathrm{SDpr}e^{2}+\mathrm{SDpos}t^{2}-2r+\mathrm{SDpre}\times \mathrm{SDpost}}$$

SDdiff denotes the standard deviation of the difference in means, SDpre indicates the standard deviation from the pre-intervention phase, and SDpost represents the standard deviation from the post-intervention phase (20). Due to the absence of reported Pearson correlation coefficients (*r*) in the original studies, we utilized *r* = 0.5 from the preceding meta-analysis (21).

If the study exclusively reported standard error (SE) or confidence intervals (CIs), they were transformed into standard deviation (SD) utilizing the following formula:

$$\mathrm{SD}=\mathrm{SE}\times \sqrt{N}$$

$$\mathrm{SD}=\sqrt{N}\times \frac{\mathrm{CIupper}-\mathrm{CIlower}}{2t}$$

In this context, SE denotes standard error, SD represents standard deviation, *N* indicates the sample size of the group, CIupper refers to the higher limit of the confidence interval, CIlower signifies the lower limit of the confidence interval, and *t* corresponds to the *t* distribution with *N*–1 degrees of freedom for the appropriate confidence interval (20). The details of outcome data for each study are available in the Supplementary Table S3.

### 2.5 Risk of bias of individual studies

Two authors (J.C. and J.Y.) independently evaluated the bias risk of the included studies using the Effective Public Health Practice Project (EPHPP) Quality Assessment Tool for Quantitative Studies (22). The quality was evaluated based on selection bias, research design, confounding variables, blinding, data collecting techniques, and participant withdrawal and dropout rates, on a grading system of 1–3 (1 = strong, 2 = moderate, 3 = weak).

### 2.6 Data synthesis and analysis

All analyses were conducted in Stata software (v14.0; StataCorp, College Station, TX, USA). Given the expected heterogeneity among the studies, the random-effects model (DerSimonian and Laird approach) was utilized to analyze the effects (23). To measure the changes in outcomes, we took the difference in changes between the intervention and control groups. We used the combined mean (M) and standard deviation (SD) of the changes from both groups for this calculation. As all outcomes of interest were continuous and potentially susceptible to slight sample bias, Hedge's was selected as the effect estimate instead of Cohen's *d* (24). The polled effect sizes for Hedge's were classified as small (0 ≤ *g* ≤ 0.50), moderate (0.50 < *g* ≤ 0.80) and large (>0.80) (25).

Heterogeneity among studies was evaluated using the inconsistency index (*I*^2^), which is based on the Cochran *Q* statistic (26). The *I*^2^ statistic is the proportion of the observed variance due to the actual between-study variance. Values of 25%, 50% and 75% might be considered as low, moderate and high, respectively (27). Publication bias was assessed with Egger regression test. Sensitivity analyses were performed on the overall results by removing each study from the model once (28). Finally, meta-regression analyses were used to assess the sources of heterogeneity for each of the outcome (29).

## 3 Results

### 3.1 Study selection

**Figure 1:**
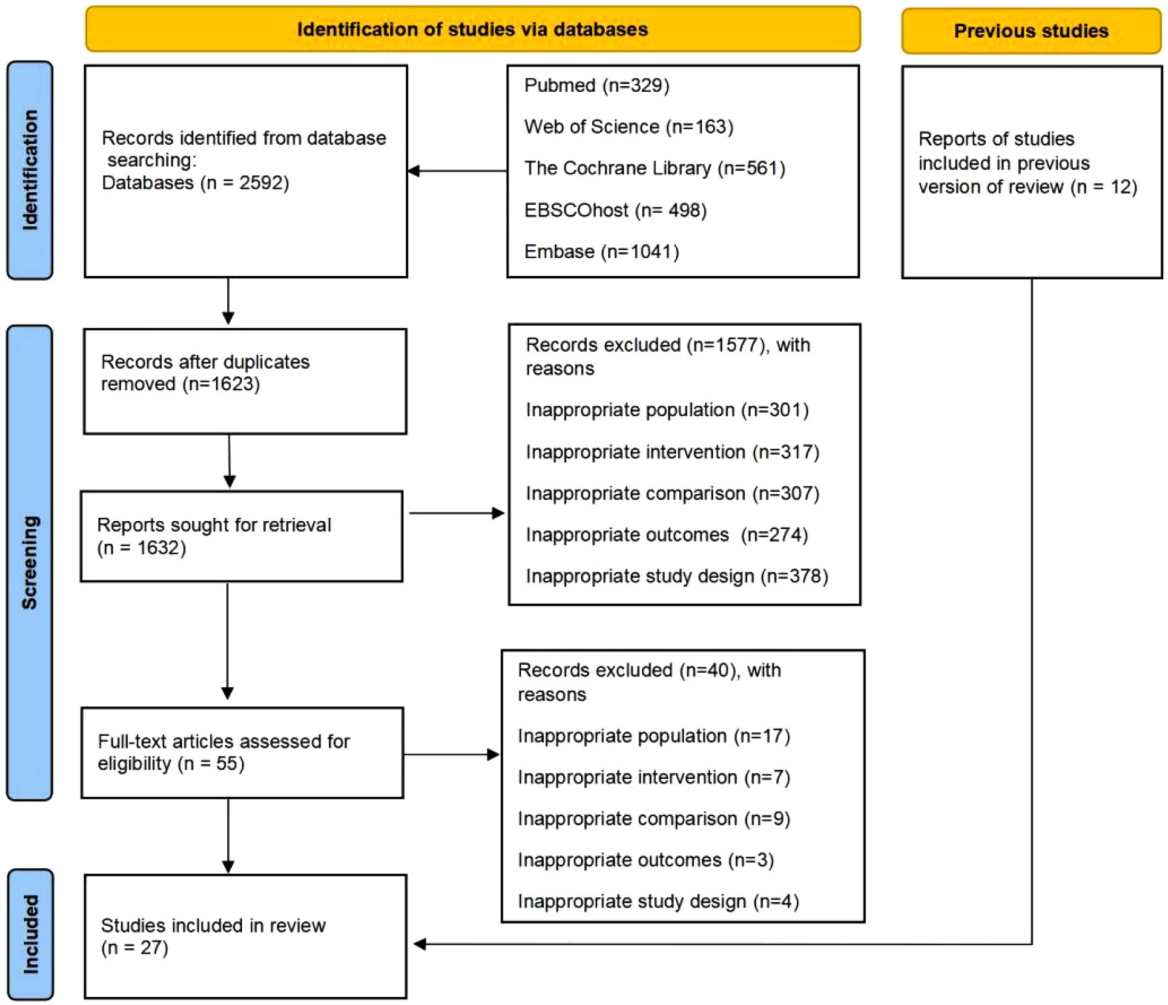
The preferred reporting items for systematic review and meta-analysis (PRISMA) flow chart of the literature search and screening.

The electronic search strategy retrieved 2,592 records (PubMed = 329; Web of Science = 163; The Cochrane Library = 561; EBSCOhost = 498; Embase = 1,041). After removing duplicate references and screening titles and abstracts, 1,632 articles were excluded. Of the remaining 1,632 articles, after full-text screening and checking the reference lists of included studies and previous reviews for additional relevant articles, 55 were read in full. The reasons for exclusion based on the full text were (1) inappropriate population (17 articles), (2) inappropriate intervention (7 articles), (3) Inappropriate comparison (9 articles), (4) Inappropriate outcomes (3 articles), and (5) Inappropriate study design (4 articles). In addition, 12 studies were identified from previously published reviews. Therefore, 27 studies were included in the final meta-analysis (12, 30–43). The PRISMA flow diagram is shown in Figure 1.

### 3.2 Study selection

Table 1 presents the characteristics of the 27 included studies. Among them, 23 articles were RCT and 4 articles were CT. Included 27 studies were from 12 countries: Germany (7 studies) (30–36), United Kingdom (6 studies) (12, 37–41), Canada (3 studies) (42–44), China (3 studies) (11, 45, 46), and 1 in each of the following countries: Belgium (47), Iran (48), Serbia (49), Singapore (50), South Africa (51), Spain (52), the Netherlands (53), USA (54). Publication dates ranged from 2000 to 2025. The final analysis included a total of 970 adults (33.8% men). Most studies included apparently healthy adults (14 studies) and overweight and/or obese adults (11 studies) (11, 12, 30–37, 39–47, 49–54), 2 studies each included hypertensive and type 2 diabetes adults (38, 48).

**Table 1:** Characteristics of included studies.

Study	Country	Design	Sample (A/B)	Age range (mean ± SD)	Men ratio (%)	Disease status	Intervention summary (mode; time; intensity; frequency; duration)	Outcomes
Banitaleb et al. (2019) (48)	Iran	RCT	14/14	A: 27 ± 7 B: 26 ± 8	0	T2D	(1) cycling; (2) 4 × 30 s; (3) all out; (4) 3/week; (5) 10 weeks.	FBG
Bhammar et al. (2017) (54)	USA	RCT	10/10	32 ± 5	100	OW/OB	(1) brisk walking; (2) 2 × 1 min; (3) 79 ± 4% HRmax; (4) 1/day; (5) 3 days.	(1) BP; (2) FBG.
Boer et al. (2014) (51)	South Africa	RCT	17/14	A: 18 ± 3.2 B: 17.4 ± 2.4	64.5	Healthy	(1) cycling; (2) 10 × 15 s; (3) 110% of VTR; (4) 2/week; (5) 15 weeks.	(1) HDL; (2) LDL; (3) FBG; (4) TG; (5) body mass; (6) BF%; (7) BP; (8) WC; (9) TC.
Boreham et al. (2000) (37)	UK	CT	12/10	A: 19.8 ± 0.3 B: 20.3 ± 0.3	0	Healthy	(1) stair climbing; (2) 2 × 1 min; (3) all out; (4) 6/week; (5) 7 weeks.	(1) HDL; (2) body mass; (3) TC.
Boreham et al. (2005) (12)	UK	CT	8/7	A: 22.1 ± 2.1 B: 21.8 ± 4	0	Healthy	(1) stair climbing; (2) 2 × 1 min; (3) all out; (4) 5/week; (5) 8 weeks.	(1) VO_2_max; (2) HDL; (3) LDL; (4) TG; (5) TC.
Chin et al. (2020) (45)	China	RCT	14/14	18–30	0	OW/OB	(1) running; (2) 12 × 1 min; (3) 70% HRR; (4) 2/week; (5) 8 weeks.	(1) HDL; (2) FBG; (3) TG.
Dogra et al. (2019) (42)	Canada	CT	10/10	24.7 ± 2.9	100	Healthy	(1) cycling; (2) 1 min; (3) all out; (4) 1/week; (5) 2weeks.	(1) HDL; (2) LDL; (3) FBG; (4) TG; (5) BP; (6) TC.
Elley et al. (2006) (38)	UK	RCT	11/12	A: 51 ± 14 B: 52 ± 9	52.2	Hypertensive	(1) brisk walking; (2) 4 × 10 min; (3) all out; (4) 2/week; (5) 6 weeks.	BP
Gillen et al. (2016) (43)	Canada	RCT	9/6	A: 27 ± 7 B: 26 ± 8	100	Healthy	(1) cycling; (2) 3 × 20 s; (3) all out; (4) 3/week; (5) 12 weeks.	(1) FBG; (2) body mass; (3) BF%;
Jabbour et al. (2017) (44)	Canada	RCT	12/12	A: 23.3 ± 2.3 B:23.1 ± 3.3	50	OW/OB	(1) cycling; (2) 6 × 6 s; (3) all out; (4) 3/week; (5) 2 weeks.	(1) VO_2_max; (2) HDL; (3) LDL; (4) FBG; (5) TG; (6) BF%; (7) BP; (8) WC; (9) TC.
Metcalfe et al. (2012) (39)	UK	RCT	15/14	A: 26 ± 3 B: 19 ± 1	44.8	Healthy	(1) cycling; (2) 2 × 10–20 s; (3) all out; (4) 3/week; (5) 6 weeks.	(1) FBG; (2) body mass; (3) BF%;
Michael et al. (2021) (40)	UK	RCT	26/10	A: 31.76 ± 1.33 B: 32 ± 2.75	0	Healthy	(1) stair climbing; (2) NA; (3) all out; (4) 5/week; (5) 8 weeks.	(1) VO_2_max; (2) HDL; (3) LDL; (4) FBG; (5) TG; (6) body mass; (7) BF%; (8) BP.
Perkin et al. (2019) (41)	UK	CT	10/10	A: 70 ± 4 B: 74 ± 5	30	Healthy	(1) self-weight; (2) 5 × 1 min; (3) all out; (4) 2/day; (5) 28 days.	(1) body mass; (2) BF%.
Reljic et al. (2020) (33)	Germany	RCT	36/29	A: 48.5 ± 10 B: 49.0 ± 9.9	44.6	OW/OB	(1) cycling; (2) 5 × 1 min; (3) 80%–95% HRpeak; (4) 2/week; (5) 12 weeks.	(1) VO_2_max; (2) HDL; (3) LDL; (4) TG; (5) BF%; (6) BP; (7) WC; (8) body mass.
Reljic et al. (2021a) (31)	Germany	RCT	32/33	A: 49.6 ± 12.3 B: 48.8 ± 13.2	43.1	OW/OB	(1) cycling; (2) 5 × 1 min; (3) 80%–95% HRpeak; (4) 2/week; (5) 12 weeks.	(1) VO_2_max; (2) HDL; (3) LDL; (4) TG; (5) BF%; (6) BP; (7) WC; (8) body mass.
Reljic et al. (2021b) (35)	Germany	RCT	29/17	A: 50.6 ± 11.3 B: 49.0 ± 15.1	43.5	OW/OB	(1) cycling; (2) 5 × 1 min; (3) 80%–95% HRpeak; (4) 2/week; (5) 12 weeks.	(1) VO_2_max; (2) HDL; (3) LDL; (4) TG; (5) BF%; (6) BP; (7) WC; (8) body mass.
Reljic et al. (2022a) (30)	Germany	RCT	26/26	A: 50.6 ± 11.3 B: 49.0 ± 15.1	46.2	OW/OB	(1) cycling; (2) 5 × 1 min; (3) 80%–95% HRpeak; (4) 2/week; (5) 12 weeks.	(1) VO_2_max; (2) HDL; (3) LDL; (4) FBG; (5) TG; (6) body mass; (7) BF%; (8) BP; (9) WC; (10) TC.
Reljic et al. (2022b) (34)	Germany	RCT	19/26	A: 50 ± 9 B: 47 ± 12	66.7	OW/OB	(1) cycling; (2) 5 × 1 min; (3) 80%–95% HRpeak; (4) 2/week; (5) 12 weeks.	(1) VO_2_max; (2) HDL; (3) LDL; (4) FBG; (5) TG; (6) body mass; (7) BF%; (8) BP; (9) WC; (10) TC.
Reljic et al. (2023) (32)	Germany	RCT	20/18	A: 50.6 ± 11.3 B: 49.0 ± 15.2	39.5	Healthy	(1) cycling; (2) 5 × 1 min; (3) 80%–95% HRpeak; (4) 2/week; (5) 12 weeks.	(1) VO_2_max; (2) HDL; (3) LDL; (4) FBG; (5) TG; (6) body mass; (7) BF%; (8) BP; (9) WC; (10) TC.
Schubert et al. (2017) (52)	Spain	RCT	12/6	28.8	44.4	OW/OB	(1) cycling; (2) (2) 3–5 × 20 s; (3) all out; (4) 3/week; (5) 4 weeks.	(1) BM; (2) BF%; (3) WC; (4) TC.
Scoubeau et al. (2023) (47)	Belgium	RCT	14/14	A: 23.1 ± 1.3 B: 24.0 ± 3.9	57.1	Healthy	(1) self-weight; (2) 4 × 30 s; (3) all out; (4) 3/week; (5) 8 weeks.	(1) VO_2_max; (2) body mass; (3) BF%.
Stojanović et al. (2021) (49)	Serbia	RCT	86/82	A: 75.7 ± 8.9 B: 74.5 ± 8.2	0	Healthy	(1) elastic band rAistance training; (2) NA; (3) all out; (4) 2/week; (5) 12 weeks.	(1) HDL; (2) LDL; (3) FBG; (4) TG.
Venegas-Carro et al. (2023) (36)	Germany	RCT	16/15	A: 24.5 ± 3.6 B:24.8 ± 3.1	51.6	Healthy	(1) running; (2) (2) 5–8 × 30 s; (3) all out; (4) 3/week; (5) 6 weeks.	VO_2_max
Wanders et al. (2021) (53)	the Netherlands	RCT	14/14	A: 75.7 ± 8.9 B: 74.5 ± 8.2	47.3	OW/OB	(1) cycling; (2) NA; (3) 50%–70% HRmax; (4) 1/week; (5) 4 weeks.	(1) HDL; (2) LDL; (3) TG; (4) TC.
Wong et al. (2024) (50)	Singapore	RCT	11/8	A: 24.0 ± 2.8 B: 24.6 ± 1.7	42.1	Healthy	(1) cycling; (2) 20 s; (3) RPEmax; (4) 5/week; (5) 6 weeks.	(1) VO_2_max; (2) FBG; (3) TG; (4) body mass; (5) BF%; (6) BP.
Yin et al. (2024) (46)	China	RCT	14/15	A: 22.1 ± 2.1 B: 21.8 ± 3	48.3	Healthy	(1) cycling; (2) 3 × 30 s; (3) all out; (4) 3/week; (5) 6 weeks.	VO_2_max
Zhou et al. (2025) (11)	China	RCT	14/13	A: 21.08 ± 1.32 B: 22.14 ± 1.88	48.1	OW/OB	(1) stair climbing; (2) NA; (3) all out; (4) 4/week; (5) 12 weeks.	(1) VO_2_max; (2) body mass; (3) BF%.

RCT, randomized controlled trial; CT, controlled trial; AS, exercise snacks; B, control; OW/OB, Overweight/obese; T2D, Type 2 Diabetes; NA, not available, RPE, rating of perceived exertion; HRpeak, heart rate peak; VT, ventilatory threshold; WC, waist circumference; BF%, body fat percentage; SBP, systolic blood pressure; DBP, diastolic blood pressure; VO_2_max, maximal oxygen uptake; FBG, fasting blood glucose; TC, total cholesterol; LDL, low-density lipoprotein; HDL, high-density lipoprotein; TG, triglycerides.

There was a large variation in the content of the ES interventions from brisk walking, running and stair climbing, self-weight and brisk walking, running and stair climbing, to more extensive programmes such as cycling. 16 research included cycling as the intervention, with 552 individuals (46.2% male) with a mean age of 30.67 years (30–35, 39, 42–44, 46, 48, 50–53). The average length of the intervention was 8.69 weeks. Stair climbing served as the intervention strategy in four investigations, including 100 individuals (13% male) with a mean age of 23.87 years (11, 12, 37, 40). The average length of the intervention was 8.75 weeks. The remaining seven trials included running (36, 45), brisk walking (38, 54), bodyweight exercises (41, 47), and elastic band resistance training as the intervention modalities (49). Three hundred eighteen people were enlisted. 18.87 of the individuals were male. A total of 318 participants, with 18.87% being males, had a mean age of 47.78 years.

### 3.3 Risk of bias within studies

The EPHPP checklist was used to evaluate all of the included meta-analyses. Out of the 27 studies, 4 were rated as having a high quality, 11 were rated as having a moderate quality, and 12 were rated as having a low quality. The primary flaws were a lack of blinding of participants and assessors, as well as selection bias. In the supplementary document, Supplementary Table S4 contains additional information.

### 3.4 Results of meta-analysis

**Figure 2:**
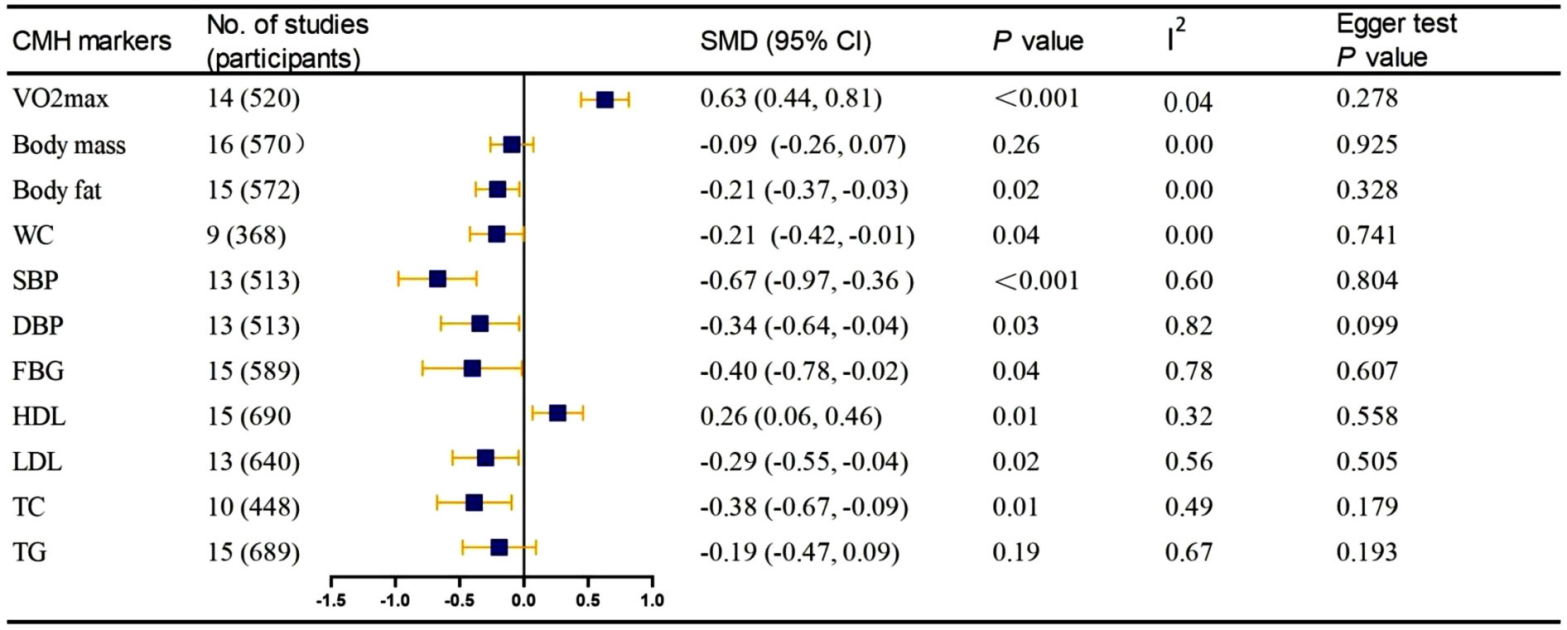
Synthesis of pooled results.

Figure 2 presents a comprehensive overview of the primary attributes of each meta-analysis, including sample information, effect size, heterogeneity and Egger test of studies. The results of meta-analysis shows that compared with the control conditions, ES interventions were associated with significant reductions in BF% [SMD (standard mean difference) = −0.205; 95% CI: −0.375, −0.036; *P* = 0.018; *I*^2^ = 0.00%], WC (SMD = −0.212; 95% CI: −0.420, −0.004; *P* = 0.045; *I*^2^ = 0.00%), SBP (SMD = −0.672; 95% CI: −0.976, −0.369; *P* < 0.001; *I*^2^ = 59.80%), DBP (SMD = −0.341; 95% CI: −0.646, −0.037; *P* = 0.028; *I*^2^ = 82.30%), FBG (SMD = −0.403; 95% CI: −0.789, −0.016; *P* = 0.041; *I*^2^ = 77.60%), LDL (SMD = −0.299; 95% CI: −0.555, −0.042; *P* = 0.023; *I*^2^ = 55.70%), and TC (SMD = −0.387; 95% CI: −0.677, −0.096; *P* = 0.009; *I*^2^ = 48.50%). The meta-analysis found a significant improvement effect of ES intervention increases in VO_2_max (SMD = 0.63; 95% CI: 0.445, 0.815; *P* < 0.001; *I*^2^ = 4.10%) and HDL (SMD = 0.263; 95% CI: 0.066, 0.459; *P* = 0.009; *I*^2^ = 32.10%). However, the meta-analysis found no statistically significant differences of ES on body mass (SMD = −0.094; 95% CI: −0.261, 0.072; *P* = 0.267; *I*^2^ = 0.00%) and TG (SMD = −0.193; 95% CI: −0.478–0.093; *P* = 0.187; *I*^2^ = 67.10%). Supplementary Figures S1–S11 demonstrates the effectiveness of ES on each of the 11 outcome indicators.

In the Egger test, the evidence for risk of publication bias was not found in the meta-analysis for all CMH marks. In the sensitivity analysis with each study deleted once from the model, the results remained the same across all deletions (Supplementary Figures S12–S22).

### 3.5 Results of meta-regression

**Figure 3:**
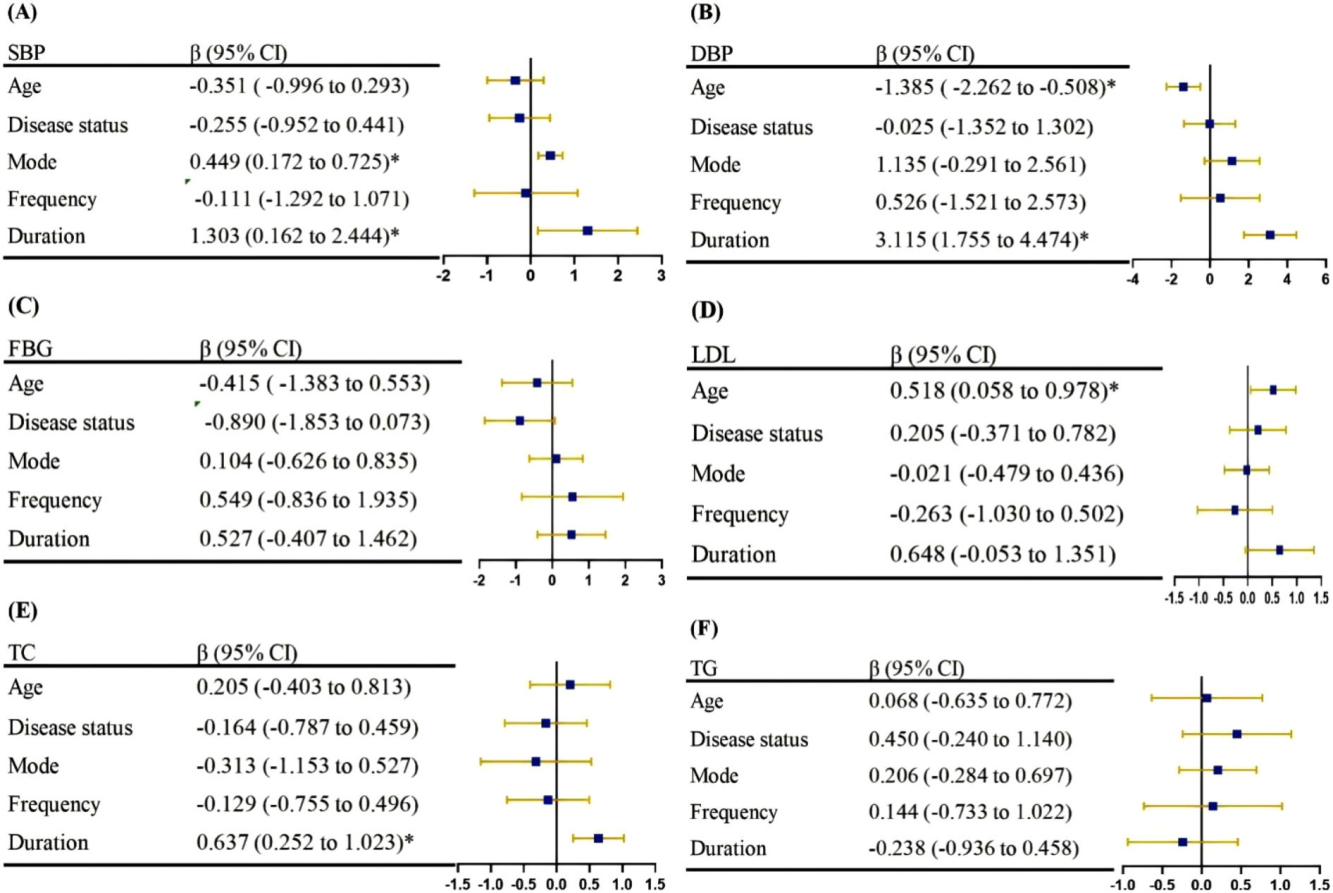
The results of meta-regression in **(A)** systolic blood pressure, **(B)** diastolic blood pressure, **(C)** fasting blood glucose, **(D)** low density lipoprotein, **(E)** total cholesterol, **(F)** triglycerid.

For results where significant heterogeneity exists, Figure 3 shows the β-coefficients and CIs for the meta-regression analysis. The results of meta-regression reveal that age could predict the improvements observed in DBP [β (95%CI): −1.385 (−2.262, −0.508)] and LDL [β (95%CI): 0.518 (0.058, 0.978)]. Mode of ES could predict the improvements observed in SBP [β (95%CI): 0.449 (0.172, 0.725)]. Additionally, duration of ES was associated with SBP [β (95%CI): 1.303 (0.162–2.444)], DBP [β (95%CI): 3.115 (1.755–4.474)] and TC [β (95%CI): 0.637 (0.252–1.023)].

## 4 Discussion

Results suggest that exercise snacks (ES) are effective and time-efficient interventions to improve cardiometabolic health (CMH) in adults. Specifically, our results found that ES beneficially influenced VO_2_max, BF%, WC, SBP, DBP, FBG, HDL, LDL. and TC, compare to control group. Unfortunately, the results show that ES cannot improve body mass and TG.

### 4.1 Effects of ES on Vo_2_max in adults

How to efficiently boost the impact of sedentary people's VO_2_max has been a topic of increasing attention among researchers and practitioners (55). Previous meta-analyses have shown that HIIT can improve cardiorespiratory fitness in various populations (16, 55, 56). However, few studies have explored the relationship between exercise snacks (ES) and VO_2_max. The potential for exercise snacks to improve VO_2_max was demonstrated in an RCT study involving Fifteen women (18.8 ± 0.7 years) who were randomly assigned to control or stair climbing groups (12). These results imply that Relative to controls, the stair climbing group displayed a 17.1% increase in VO_2_max. In another RCT research, Michael et al. subjected thirty-six inactive women (31.7 ± 1.4 years) to an intervention involving daily bouts of strenuous stair climbing on 5 d·wk−1 or a no-exercise control (*n* = 10) (40). For each stair-based workout snack, participants were told to safely ascend the 32.8-m climb as quickly as feasible. After the 8-week intervention, there were minor, yet substantial, improvements in VO2max as evaluated using the Multi-Stage Fitness Test (MSFT) (57).

Meanwhile, this identical result was verified in sedentary men (31, 32). Moreover, our findings further complement the existing evidence showing the benefit of conducted ES in enhancing VO_2_max. The possible mechanisms of ES to improve cardiorespiratory fitness are as follows: skeletal muscle mitochondrial density regulates substrate metabolism during high intensity exercise, with increased mitochondrial content promoting a greater reliance on fat oxidation and enhanced the oxygen utilization rate in peripheral blood (58, 59). As a result, ES could improve VO_2_max and cardiorespiratory fitness (10). In addition, Gibala et al. found that short-term high-intensity exercise and traditional endurance training might generate equal gains in muscle oxidative capacity by measuring the maximal activity of cytochrome c oxidase (COX) and COX subunits II and IV protein content (43). So, ES has an advantage in enhancing cardiovascular fitness when the energy expenditure of ES is equivalent to typical endurance training. In conclusion, this deserves additional research to explore better the optimal approaches for enhancing ES of VO_2_max.

### 4.2 Effects of ES on body composition in adults

In the last few years, developmental evidence has been accumulating, showing the beneficial impacts of ES on adults' body composition. Previous research has revealed that acute bouts of high-intensity exercise may accelerate fat decomposition by boosting the release of catecholamines, adrenaline, norepinephrine, and growth hormone (58–61). Perkin et al. observed that ES programs seem to be an effective intervention for lowering BF% and WC (41). Consistent with the literature, our meta-analysis demonstrated the beneficial effects of ES on BF% and WC compared to the control group. Nevertheless, there is still a dispute concerning the efficiency of ES for weight loss and the fat-burning mechanism of ES (62). The improvements in body composition are influenced by a range of factors, including, but not limited to, the impacts of age, gender, food, and lifestyle (63). This explains why ES had a limited effect on body fat and waist circumference in the results of the present investigation and even failed to affect body weight. Similarly, in a meta-analysis by Cao et al. that included 20 studies, results showed that compared with MICT, HIIT caused no significant differences in body weight (66). To be sure, weight loss management is a day-to-day procedure (64, 67, 68). The Institute of Medicine recommends that a weight shift of higher than five is regarded as potentially clinically important and that weight should be maintained below this minimum number for at least 1 year (65). Therefore, additional research on the long-term impacts of ES fat loss is needed in the future.

### 4.3 Effects of ES on BP in adults

PA is a crucial intervention for preventing hypertension and enhancing long-term survival (69). Prior research has indicated a correlation between ES and blood pressure levels in adults. For example, Jabbour et al. showed that systolic blood pressure in overweight, obese adult participants was significantly reduced 24 h after a 6-week ES intervention compared to baseline and the control group, with these effects remaining significant at 72 h and 2 weeks post-intervention (44). Reljic et al. studied 65 inactive obese persons and found that after cycling biweekly for 12 weeks, participants in the intervention group exhibited modest enhancements in mean arterial blood pressure relative to the control group (33). This aligns with our findings. Our meta-analysis demonstrated a beneficial effect of ES on SBP and DBP in comparison to the Control group. This aligns with the results of prior research. Consequently, the existing evidence is unequivocal regarding the beneficial effect of ES on BP.

### 4.4 Effects of ES on FBG in adults

Exercise effectively reduces fasting blood glucose levels and improves insulin resistance, with the intensity of training being a crucial factor (70, 71). In a recent systematic review and meta-analysis, Xing and his colleagues compared the effects of different exercise interventions on patients with type 2 diabetes (72). The results showed that moderate-intensity exercise appears to be an effective way to improve HbA1c, TG levels, and LDL, while high-intensity exercise is the best option for improving FBG. A crossover study by Mezghani et al. involving 25 pre-diabetic patients demonstrated that post-exercise insulin and blood glucose levels significantly decreased at an exercise intensity of 70% compared to 50% and 60% (*p* ≤ 0.001) (73). Fasting blood glucose levels, which ranged from 110 to 115 mg/dl, showed a significant reduction at both 30- and 60 min post-exercise (*p* ≤ 0.001). This suggests that high-intensity exercise is generally more effective in lowering fasting glucose levels and improving insulin resistance. Despite these findings, no systematic review and meta-analysis have previously investigated the impact of exercise sessions (ES) on fasting blood glucose (FBG). Previous meta-analyses have shown that high-intensity interval training (HIIT) significantly reduces insulin resistance compared to control (CON) and continuous training (CT) groups (74). High-intensity exercise recruits a larger proportion of muscle fibers and enhances the expression and transport of glucose transporter protein 4 (GLUT4) to the surface of skeletal muscle cells (75). This process improves insulin transport efficiency and sensitivity, leading to lower blood glucose levels and reduced insulin secretion (76, 77). The findings indicate that exercise sessions significantly enhance fasting blood glucose levels, highlighting their role in diabetes management. However, the changes in fasting blood glucose observed in this model were minimal. Therefore, subsequent studies should explore the clinical implications of exercise sessions on fasting blood glucose.

### 4.5 Effects of ES on lipid metabolism in adults

Sedentary adults frequently experience lipid metabolism disorders, resulting in a heightened risk of cardiovascular disease (CVD) (78, 79). The findings of our research indicate that ES, as a highly time-efficient training protocol, significantly impacted HDL (SMD = 0.263), LDL (SMD = −0.299), and TC (SMD = −0.387) when compared to the control group but did not affect TG (SMD = −0.193). Our findings align with those of Reljic et al, who similarly reported no improvement in TG with ES (33). Chin et al. observed that alterations in TG levels were significantly reduced in overweight and obese individuals after an 8-week exercise intervention involving running (45). This contradicts our findings; however, we can explain. The studies included in our meta-analysis primarily focused on cycling and stair climbing. Cycling predominantly engages the lower extremities and is mainly dependent on carbohydrate oxidation. Running, in contrast, promotes lipolysis through elevated levels of adrenaline, noradrenaline, and growth hormone, resulting in potentially greater fat utilization during running compared to cycling for equivalent exercise duration (80, 81). Secondly, research indicates that running engages a greater muscle mass than cycling, resulting in elevated energy expenditure and higher maximal oxidation rates (82, 83). This difference may be attributed to variations in training styles.

### 4.6 Limitations and future research

To the best of our knowledge, it is the first systematic review and meta-analysis examining the impact of ES (≤1 min of high-intensity exercise) on CMH. However, many limitations must be acknowledged.

First, this systematic review and meta-analysis encompasses 27 peer-reviewed studies with a total of 970 participants. The limited sample size and the low (12/27) or moderate (11/27) quality of the majority of the included studies led to potential publication bias in the findings, restricted generalizability of the results, and potential overestimation of effects. We thoroughly explored mainstream databases to mitigate this risk and employed Egger's test to confirm the absence of such bias. Subsequent research should employ designs with higher sample numbers to enhance the precision and external validity of the results.

Secondly, substantial heterogeneity was evident in the outcome measures (e.g., systolic blood pressure, fasting blood glucose), likely stemming from differences in intervention protocols and sample characteristics. Although random-effects models and meta-regression analysis were employed to identify potential sources of heterogeneity, further subgroup analyses were hindered by the limited number of studies included. Future research should focus on examining the effect sizes (ES) of various intervention strategies and prioritize large-scale studies. The ultimate goal of such efforts would be to elucidate the impact of these variables more thoroughly.

Finally, the duration of the included ES therapies ranged from 8 to 12 weeks, with one trial extended to 15 weeks. Given the need to achieve optimal efficacy in clinical and public health practice, the National Institute for Health and Care Excellence (NICE) emphasizes the use of controlled trials with at least 12 months of follow-up. Therefore, future large-scale randomized controlled trials should explore long-term studies to assess the sustainability of efficacy.

## 5 Conclusion

This systematic review and meta-analysis investigated the impact of ES treatments on CMH in adults, spanning a wide variety of cardiometabolic markers such as VO_2_max, body mass, BF%, WC, SBP, DBP, FBG, HDL, LDL, TC, and TG. We adopted an algorithmic strategy for standardizing and integrating the varied data, enabling a unified comparison of the influence of ES on CMH in people. By assessing 27 trials encompassing 11 distinct CMH outcomes, we found that ES significantly improved nine cardiometabolic indicators VO_2_max, BF%, WC, SBP, DBP, FBG, HDL, LDL, and TC but not body mass and TG relative to no intervention.

As the first systematic review and meta-analysis to evaluate the role of ES interventions in CMH for adults, this study fills a crucial gap in understanding how these programs can be integrated into clinical practice. The findings provide robust evidence for clinicians and public health professionals, emphasizing the potential of ES as a valuable tool for improving key cardiometabolic indicators. Clinicians may consider incorporating ES into routine care protocols to enhance patient outcomes, particularly for individuals with risk factors for cardiovascular and metabolic diseases. For public health practitioners, these findings underscore the need to advocate for ES programs as part of community health strategies to prevent and manage CMH.
