# Supplementary material for: The effectiveness of exercise snacks as a time-efficient treatment for improving cardiometabolic health in adults: a systematic review and meta-analysis
Source: Front Cardiovasc Med. 2025 Aug 13;12:1643153. doi: 10.3389/fcvm.2025.1643153 (PMC12380701; doi:10.3389/fcvm.2025.1643153)
Supplement: Supplementary file 1 [file Datasheet1.pdf]

Supplementary Material

Table S1. The PRISMA checklist

Table S2. The details of full search strategy for systematic review and meta analysis

Table S3. The details of outcome data for each study

Table S4. Risk of bias within studies

Figure S1. The forest plot showing the effects sizes (SMD) of ES interventions on VO_2max

Figure S2. The forest plot showing the effects sizes (SMD) of ES interventions on body mass

Figure S3. The forest plot showing the effects sizes (SMD) of ES interventions on BF%

Figure S4. The forest plot showing the effects sizes (SMD) of ES interventions on WC

Figure S5. The forest plot showing the effects sizes (SMD) of ES interventions on SBP

Figure S6. The forest plot showing the effects sizes (SMD) of ES interventions on DBP

Figure S7. The forest plot showing the effects sizes (SMD) of ES interventions on FBG

Figure S8. The forest plot showing the effects sizes (SMD) of ES interventions on HDL

Figure S9. The forest plot showing the effects sizes (SMD) of ES interventions on LDL

Figure S10. The forest plot showing the effects sizes (Hedge' s g) of ES interventions on TC

Figure S11. The forest plot showing the effects sizes (SMD) of ES interventions on TG

Figure S12. The forest plot showing the sensitivity analysis of ES interventions on VO_2max for each study

Figure S13. The forest plot showing the sensitivity analysis of ES interventions on body mass for each study

Figure S14. The forest plot showing the sensitivity analysis of ES interventions on BF% for each study

Figure S15. The forest plot showing the sensitivity analysis of ES interventions on WC for each study

Figure S16. The forest plot showing the sensitivity analysis of ES interventions on SBP for each study

Figure S17. The forest plot showing the sensitivity analysis of ES interventions on DBP for each study

Figure S18. The forest plot showing the sensitivity analysis of ES interventions on FBG for each study

Figure S19. The forest plot showing the sensitivity analysis of ES interventions on HDL for each study

Figure S20. The forest plot showing the sensitivity analysis of ES interventions on LDL for each study

Figure S21. The forest plot showing the sensitivity analysis of ES interventions on TC for each study

Figure S22. The forest plot showing the sensitivity analysis of ES interventions on TG for each study

Table S1. The PRISMA checklist

Section and Topic	Item #	Checklist item	Location where item is reported
**TITLE**			
Title	1	Identify the report as a systematic review.	
**ABSTRACT**			
Abstract	2	See the PRISMA 2020 for Abstracts checklist.	
**INTRODUCTION**			
Rationale	3	Describe the rationale for the review in the context of existing knowledge.	
Objectives	4	Provide an explicit statement of the objective(s) or question(s) the review addresses.	
**METHODS**			
Eligibility criteria	5	Specify the inclusion and exclusion criteria for the review and how studies were grouped for the syntheses.	
Information sources	6	Specify all databases, registers, websites, organisations, reference lists and other sources searched or consulted to identify studies. Specify the date when each source was last searched or consulted.	
Search strategy	7	Present the full search strategies for all databases, registers and websites, including any filters and limits used.	
Selection process	8	Specify the methods used to decide whether a study met the inclusion criteria of the review, including how many reviewers screened each record and each report retrieved, whether they worked independently, and if applicable, details of automation tools used in the process.	
Data collection process	9	Specify the methods used to collect data from reports, including how many reviewers collected data from each report, whether they worked independently, any processes for obtaining or confirming data from study investigators, and if applicable, details of automation tools used in the process.	
Data items	10a	List and define all outcomes for which data were sought. Specify whether all results that were compatible with each outcome domain in each study	

Section and Topic	Item #	Checklist item	Location where item is reported
		were sought (e.g. for all measures, time points, analyses), and if not, the methods used to decide which results to collect.	
	10b	List and define all other variables for which data were sought (e.g. participant and intervention characteristics, funding sources). Describe any assumptions made about any missing or unclear information.	
Study risk of bias assessment	11	Specify the methods used to assess risk of bias in the included studies, including details of the tool(s) used, how many reviewers assessed each study and whether they worked independently, and if applicable, details of automation tools used in the process.	
Effect measures	12	Specify for each outcome the effect measure(s) (e.g. risk ratio, mean difference) used in the synthesis or presentation of results.	
Synthesis methods	13a	Describe the processes used to decide which studies were eligible for each synthesis (e.g. tabulating the study intervention characteristics and comparing against the planned groups for each synthesis (item #5)).	
	13b	Describe any methods required to prepare the data for presentation or synthesis, such as handling of missing summary statistics, or data conversions.	
	13c	Describe any methods used to tabulate or visually display results of individual studies and syntheses.	
	13d	Describe any methods used to synthesize results and provide a rationale for the choice(s). If meta-analysis was performed, describe the model(s), method(s) to identify the presence and extent of statistical heterogeneity, and software package(s) used.	
	13e	Describe any methods used to explore possible causes of heterogeneity among study results (e.g. subgroup analysis, meta-regression).	
	13f	Describe any sensitivity analyses conducted to assess robustness of the synthesized results.	
Reporting bias assessment	14	Describe any methods used to assess risk of bias due to missing results in a synthesis (arising from reporting biases).	
Certainty assessment	15	Describe any methods used to assess certainty (or confidence) in the body of evidence for an outcome.	

Section and Topic	Item #	Checklist item	Location where item is reported
**RESULTS**			
Study selection	16a	Describe the results of the search and selection process, from the number of records identified in the search to the number of studies included in the review, ideally using a flow diagram.	
	16b	Cite studies that might appear to meet the inclusion criteria, but which were excluded, and explain why they were excluded.	
Study characteristics	17	Cite each included study and present its characteristics.	
Risk of bias in studies	18	Present assessments of risk of bias for each included study.	
Results of individual studies	19	For all outcomes, present, for each study: (a) summary statistics for each group (where appropriate) and (b) an effect estimate and its precision (e.g. confidence/credible interval), ideally using structured tables or plots.	Table S3
Results of syntheses	20a	For each synthesis, briefly summarise the characteristics and risk of bias among contributing studies.	
	20b	Present results of all statistical syntheses conducted. If meta-analysis was done, present for each the summary estimate and its precision (e.g. confidence/credible interval) and measures of statistical heterogeneity. If comparing groups, describe the direction of the effect.	
	20c	Present results of all investigations of possible causes of heterogeneity among study results.	
	20d	Present results of all sensitivity analyses conducted to assess the robustness of the synthesized results.	
Reporting biases	21	Present assessments of risk of bias due to missing results (arising from reporting biases) for each synthesis assessed.	
Certainty of evidence	22	Present assessments of certainty (or confidence) in the body of evidence for each outcome assessed.	
**DISCUSSION**			

Section and Topic	Item #	Checklist item	Location where item is reported
Discussion	23a	Provide a general interpretation of the results in the context of other evidence.	
	23b	Discuss any limitations of the evidence included in the review.	
	23c	Discuss any limitations of the review processes used.	
	23d	Discuss implications of the results for practice, policy, and future research.	
**OTHER INFORMATION**			
Registration and protocol	24a	Provide registration information for the review, including register name and registration number, or state that the review was not registered.	
	24b	Indicate where the review protocol can be accessed, or state that a protocol was not prepared.	
	24c	Describe and explain any amendments to information provided at registration or in the protocol.	
Support	25	Describe sources of financial or non-financial support for the review, and the role of the funders or sponsors in the review.	
Competing interests	26	Declare any competing interests of review authors.	
Availability of data, code and other materials	27	Report which of the following are publicly available and where they can be found: template data collection forms; data extracted from included studies; data used for all analyses; analytic code; any other materials used in the review.	

Table S2. The details of full search strategy for systematic review and meta analysis

Database	Search Strategy
PubMed	("exercise snack"[All Fields] OR "movement breaks"[All Fields] OR "physical activity breaks"[All Fields] OR (("exercise"[MeSH Terms] OR "exercise"[All Fields] OR "exercises"[All Fields] OR "exercise therapy"[MeSH Terms] OR ("exercise"[All Fields] AND "therapy"[All Fields]) OR "exercise therapy"[All Fields] OR "exercising"[All Fields] OR "exercise s"[All Fields] OR "exercised"[All Fields] OR "exerciser"[All Fields] OR "exercisers"[All Fields]) AND ("burst"[All Fields] OR "bursting"[All Fields] OR "bursts"[All Fields])) OR "high-intensity interval training"[All Fields] OR "low-volume high-intensity interval training"[All Fields]) AND ("waist circumference"[All Fields] OR "body weight"[All Fields] OR "fasting blood glucose"[All Fields] OR "total cholesterol"[All Fields] OR "systolic blood pressure"[All Fields] OR "diastolic blood pressure"[All Fields] OR "low-density lipoprotein"[All Fields] OR "high-density lipoprotein"[All Fields] OR "maximal oxygen uptake"[All Fields] OR "triglycerides"[All Fields] OR "body fat percentage"[All Fields]) AND ("clinical trial"[Publication Type] OR "randomized controlled trial"[Publication Type]) AND "English"[Language]
Web of Science	TS=("exercise snack" OR "movement breaks" OR "physical activity breaks" OR "exercise bursts" OR "high-intensity interval training" OR "low-volume high-intensity interval training") AND TS=("waist circumference" OR "body weight" OR "fasting blood glucose" OR "total cholesterol" OR "systolic blood pressure" OR "diastolic blood pressure" OR "low-density lipoprotein" OR "high-density lipoprotein" OR "maximal oxygen uptake" OR "triglycerides" OR "body fat percentage")

	AND TS=("clinical trial" OR "randomized controlled trial")
	AND LA=(English)
The Cochrane Library	#1 ("exercise snacks"):ab,ti,kw
	#2 ("movement breaks"):ab,ti,kw
	#3 ("physical activity breaks"):ab,ti,kw
	#4 ("exercise bursts"):ab,ti,kw
	#5 ("high-intensity interval training"):ab,ti,kw
	#6 ("low-volume high-intensity interval training"):ab,ti,kw
	#7 #1 OR #2 OR #3 OR #4 OR #5 OR #6
	#8 ("waist circumference"):ab,ti,kw
	#9 ("body weight"):ab,ti,kw
	#10 ("fasting blood glucose"):ab,ti,kw
	#11 ("total cholesterol"):ab,ti,kw
	#12 ("systolic blood pressure"):ab,ti,kw
	#13 ("diastolic blood pressure"):ab,ti,kw
	#14 ("low-density lipoprotein"):ab,ti,kw

	#15 ("high-density lipoprotein"):ab,ti,kw
	#16 ("maximal oxygen uptake"):ab,ti,kw
	#17 ("triglycerides"):ab,ti,kw
	#18 ("body fat percentage"):ab,ti,kw
	#19 #8 OR #9 OR #10 OR #11 OR #12 OR #13 OR #14 OR #15 OR #16 OR #17 OR #18
	#20 ("clinical trial"):ab,ti,kw
	#21 ("randomized controlled trial"):ab,ti,kw
	#22 #20 OR #21
	#23 "English":la
	#7 AND #19 AND #22 AND #23
EBSCOhost	S1 ((DE "EXERCISE" OR DE "ANAEROBIC exercises" OR DE "CIRCUIT training" OR DE "PLYOMETRICS" OR DE "RUNNING" OR DE "TREADMILL exercise" OR DE "ELLIPTICAL trainers" OR DE "PHYSICAL training & conditioning") OR (DE "INTERVAL training")) AND (DE "HIGH-intensity interval training" OR DE "INTERVAL training")
	S2 TI bout* or burst* or snack* or snacking or accumulated or dispersed

S3 TI (((high or vigorous) N2 intens*) and (training or activit*)) N2 (brief or interval* or intermittent or sporadic or cycle* or hourly or short or dispersed)

S4 TI ((high or vigorous) N2 intens* N3 (bout* or burst* or snack* or snacking or breaks))

S5 TI ((sprint* or circuit-based or all-out or plyometric* or resistance) N2 (bout* or burst* or brief or snack* or snacking or breaks))

S6 TI ((sprint or circuit-based or all-out or plyometric or resistance) N2 (training or activit*) N3 (brief or interval* or intermittent or sporadic or cycle* or hourly or short or dispersed))

S7 TI ((extreme or strength or strenuous) N2 (training or activit*) N3 (brief or interval* or intermittent or sporadic or cycle* or hourly or short or dispersed))

S8 TI ((exercise or bicycle or cycle or weight-lifting) N2 (bout* or burst* or snack* or snacking))

S9 TI ((extreme or strength or strenuous) N2 (bout* or burst* or snack* or snacking))

S10 TI (((exercise or bicycle or weight-lifting or running) N2 (brief or interval* or intermittent or sporadic or cycle* or hourly or short or dispersed)) and (high or vigorous or extreme or strenuous or intens*))

S11 TI ((extreme or strength or strenuous) N2 (bout* or burst* or snack* or snacking))

S12 TS=("waist circumference" OR "body weight" OR "fasting blood glucose" OR "total cholesterol" OR "systolic blood pressure" OR "diastolic blood pressure" OR "low-density lipoprotein" OR "high-density lipoprotein" OR "maximal oxygen uptake" OR "triglycerides" OR "body fat percentage")

S13 S3 OR S4 OR S5 OR S6 OR S7 OR S8 OR S9 OR S10 OR S11

S14 ("clinical trial" OR "randomized controlled trial")

S15 S12 AND S13 AND S14

	S16 Narrow by Language: - English
	S17 S15 AND S16
Embase 514	1 'exercise snacks' OR 'movement breaks' OR 'physical activity breaks' OR 'exercise bursts' OR 'high-intensity interval training' OR 'low-volume high-intensity interval training'<br>2 'waist circumference' OR 'body weight' OR 'fasting blood glucose' OR 'total cholesterol' OR 'systolic blood pressure' OR 'diastolic blood pressure' OR 'low-density lipoprotein' OR 'high-density lipoprotein' OR 'maximal oxygen uptake' OR 'triglycerides' OR 'body fat percentage'<br>3 'clinical trial' OR 'randomized controlled trial'<br>4 'English'<br>5 1 AND 2 AND 3 AND 4

Table S3. The details of outcome data for each study

Study	Outcome	Group	$Mean_{pre}$	SD_{pre}	$mean_{post}$	SD_{post}	N	$Mean_{change}$	SD_{change}
Banitaleb et al.(2019)	fasting glucose	ES	214.6	27.67	163.86	71.47	14	-50.74	62.41806549
		CON	200.86	46.88	190.5	59.7	14	-10.36	54.4342576
Bhammar et al.(2017)	SBP	ES	122	8	104	13	5	-18	11.35781669
		CON	123	8	128	14	5	5	12.16552506
	DBP	ES	76	11	76	11	5	0	11
		CON	79	10	77	12	5	-2	11.13552873
	fasting glucose	ES	5.4	1.2	5.2	0.8	5	-0.2	1.058300524
		CON	5.2	0.7	5.6	1.1	5	0.4	0.964365076
Boer et al.(2014)	HDL	ES	59.3	16.9	55.9	15.6	17	-3.4	16.28895331
		CON	54.9	13.5	59.4	11.4	14	4.5	12.58213019
	LDL	ES	105.2	12.4	95.6	9.3	17	-9.6	11.17720895
		CON	92.6	23.5	96	21.2	14	3.4	22.43858284
	fasting glucose	ES	86	7.6	85	7.1	17	-1	7.362744054
		CON	86	6.1	85	4.1	14	-1	5.386093204
	triglycerides	ES	79.2	22.2	70.8	16.7	17	-8.4	20.0247347
		CON	96.6	75.6	95	85.6	14	-1.6	81.06392539
	body mass	ES	76.8	18.3	76	19.1	17	-0.8	18.71282982
		CON	79.3	14.7	80	14.4	14	0.7	14.5523194
	%body fat	ES	34.2	6.9	30.4	7	17	-3.8	6.950539547
		CON	32	7.1	32	7	14	0	7.050531895
	SBP	ES	124	10	113	8	17	-11	9.16515139
		CON	118	10	119	10	14	1	10
	DBP	ES	74	7	77	8	17	3	7.549834435

		CON	72	5	72	5	14	0	5
	WC	ES	95.8	13.1	91.5	13.1	17	-4.3	13.1
		CON	95	8.8	95.9	8.2	14	0.9	8.515867542
	TC	ES	169.8	25.2	154.8	22.9	17	-15	24.13234344
		CON	169.6	29.7	171.9	25.8	14	2.3	27.95478492
Boreham et al.(2000)	HDL	ES	1.24	0.09	1.48	0.14	12	0.24	0.122882057
		CON	1.64	0.11	1.67	0.16	10	0.03	0.141774469
	body mass	ES	57.3	2.1	57.3	2	12	0	2.051828453
		CON	57.8	2	57.8	2.2	10	0	2.107130751
	TC	ES	4.93	0.25	4.53	0.24	12	-0.4	0.245153013
		CON	4.66	0.18	4.58	0.3	10	-0.08	0.261533937
Boreham et al.(2005)	VO2max	ES	NA	NA	NA	NA	8	4.5	3.7
		CON	NA	NA	NA	NA	7	1	2.1
	HDL	ES	NA	NA	NA	NA	8	-0.05	0.11
		CON	NA	NA	NA	NA	7	0.09	0.27
	LDL	ES	NA	NA	NA	NA	8	-0.17	0.27
		CON	NA	NA	NA	NA	7	0.17	0.32
	triglycerides	ES	NA	NA	NA	NA	8	-0.15	0.34
		CON	NA	NA	NA	NA	7	-0.11	0.26
	TC	ES	NA	NA	NA	NA	8	-0.05	0.41
		CON	NA	NA	NA	NA	7	0.21	0.25
Chin et al.(2020)	HDL	ES	1.39	0.27	1.41	0.26	14	0.02	0.265141472
		CON	1.31	0.23	1.28	0.32	14	-0.03	0.285832119
	fasting glucose	ES	4.66	0.22	4.67	0.41	14	0.01	0.355387113
		CON	4.7	0.25	4.69	0.34	14	-0.01	0.305122926
	triglycerides	ES	1.25	0.89	0.9	0.47	14	-0.35	0.771167945

		CON	1	0.33	1.26	0.6	14	0.26	0.520480547
Dogra et al.(2019)	HDL	ES	1.3	0.3	1.3	0.3	10	0	0.3
		CON	1.4	0.4	1.3	0.4	10	-0.1	0.4
	LDL	ES	2.5	0.7	2.4	0.7	10	-0.1	0.7
		CON	2.5	1.1	2.5	0.6	10	0	0.953939201
	fasting glucose	ES	4.7	0.3	5.7	0.8	10	1	0.7
		CON	4.5	0.5	5	0.7	10	0.5	0.6244998
	triglycerides	ES	1	0.3	1.4	0.5	10	0.4	0.435889894
		CON	1.2	0.5	1.6	0.9	10	0.4	0.781024968
	SBP	ES	111.4	12.5	110.5	10.7	10	-0.9	11.70427272
		CON	117.2	11.9	110.6	15	10	-6.6	13.7153199
	DBP	ES	71.4	6	70	7.8	10	-1.4	7.073895674
		CON	74.2	6.9	71.6	6.4	10	-2.6	6.664082833
	TC	ES	4.3	0.5	4.4	0.7	10	0.1	0.6244998
		CON	4.4	0.8	4.6	0.8	10	0.2	0.8
Elley et al.(2006)	SBP	ES	NA	NA	NA	NA	31	-7.3	3.953076046
		CON	NA	NA	NA	NA	31	-5.4	2.72625934
	DBP	ES	NA	NA	NA	NA	31	1	3.953076046
		CON	NA	NA	NA	NA	31	-0.2	2.726259342
Gillen et al.(2016)	fasting glucose	ES	5.3	0.8	5.7	0.9	9	0.4	0.854400375
		CON	5.5	1.6	5.4	0.8	6	-0.1	1.385640646
	body mass	ES	72.59	11.48	72.34	11.39	14	-0.25	11.43526563
		CON	76.3	9.59	75.55	9.23	14	-0.75	9.415163302
	%body fat	ES	42.57	2.17	41.43	4.18	14	-1.14	3.620870061
		CON	42.64	4.95	44.5	2.17	14	1.86	4.297662155
Jabbour et al.(2017)	VO2max	ES	22.2	7.4	22.2	7.4	12	0	7.4

		CON	23.4	8.4	23.4	8.4	12	0	8.4
	HDL	ES	1.07	0.08	1.04	0.08	12	-0.03	0.08
		CON	1.07	0.08	1.07	0.09	12	0	0.085440037
	LDL	ES	3.82	0.08	3.75	0.08	12	-0.07	0.08
		CON	3.82	0.07	3.86	0.06	12	0.04	0.065574385
	fasting glucose	ES	4.64	0.15	4.33	0.27	12	-0.31	0.23430749
		CON	4.61	0.11	4.63	0.14	12	0.02	0.127671453
	triglycerides	ES	1.29	0.11	1.23	0.17	12	-0.06	0.149331845
		CON	1.28	0.17	1.29	0.19	12	0.01	0.180831413
	%body fat	ES	44.3	9.4	42.1	7.1	12	-2.2	8.487048957
		CON	42.3	9.4	42.8	7.4	12	0.5	8.576712657
	SBP	ES	127	3	120	0	12	-7	3
		CON	127	3	128	4	12	1	3.605551275
	DBP	ES	80	3	77	6	12	-3	5.196152423
		CON	80	3	80	4	12	0	3.605551275
	WC	ES	101.3	2.7	101.3	2.7	12	0	2.7
		CON	101.3	2.7	101.3	2.7	12	0	2.7
	TC	ES	4.89	0.22	4.79	0.19	12	-0.1	0.206639783
		CON	4.89	0.19	4.92	0.29	12	0.03	0.255147016
Metcalfe et al.(2011)	fasting glucose	ES	789	65	695	53	15	-94	59.9082632
		CON	748	108	850	93	14	102	101.3360745
	body mass	ES	84	23	83	22	9	-1	22.5166605
		CON	78	23	78	23	6	0	23
Michael et al.(2021)	VO2max	ES	24.99	0.86	26.64	0.98	26	1.65	0.92585096
		CON	25.5	1.32	26.03	1.52	10	0.53	1.430524379
	HDL	ES	1.39	0.08	1.54	0.08	26	0.15	0.08

		CON	1.58	0.06	1.64	0.06	10	0.06	0.06
	LDL	ES	3.48	0.24	3.28	0.22	26	-0.2	0.230651252
		CON	2.74	0.26	2.88	0.22	10	0.14	0.242487113
	fasting glucose	ES	5.06	0.06	4.86	0.09	26	-0.2	0.079372539
		CON	5	0.12	5.03	0.07	10	0.03	0.104403065
Perkin et al.(2019)	body mass	ES	69	10	69	10	17	0	10
		CON	70.5	11.2	70.3	11.4	17	-0.2	11.30132736
	%body fat	ES	34	7	33.7	7	17	-0.3	7
		CON	35.1	7.4	35.2	6.8	17	0.1	7.118988692
Reljic et al.(2020)	VO2max	ES	22.5	6.5	26	6.5	36	3.5	6.5
		CON	23.1	8	22.5	8.7	29	-0.6	8.371977066
	HDL	ES	47	10	48	11	36	1	10.53565375
		CON	55	14	55	14	29	0	14
	LDL	ES	148	30	141	30	36	-7	30
		CON	145	38	141	33	29	-4	35.76310948
	triglycerides	ES	137	57	131	49	36	-6	53.45091206
		CON	134	67	124	57	29	-10	62.6019169
	%body fat	ES	43.7	7.7	41.7	8	36	-2	7.854298186
		CON	44.3	8.7	42.9	9.3	29	-1.4	9.014987521
	SBP	ES	147	17	135	15	32	-12	16.09347694
		CON	137	11	136	12	33	-1	11.53256259
	DBP	ES	96	10	86	10	36	-10	10
		CON	90	9	87	10	29	-3	9.539392014
	WC	ES	120.5	18.9	113	18	30	-7.5	18.46645608
		CON	110.7	11.1	109.2	11.8	19	-1.5	11.4660368
	body mass	ES	121.9	28.9	116.6	28.1	36	-5.3	28.50841981

		CON	109.4	18.7	105.7	19.7	29	-3.7	19.21952133
	VO2max	ES	21.9	5.3	25.1	5.4	32	3.2	5.350700889
		CON	21	7.3	20.7	7.4	33	-0.3	7.350510186
	HDL	ES	48	10	49	11	32	1	10.53565375
		CON	54	12	54	13	33	0	12.52996409
	LDL	ES	148	28	147	31	32	-1	29.61418579
		CON	146	33	142	29	33	-4	31.19294792
	triglycerides	ES	137	59	133	45	32	-4	53.3947563
		CON	151	80	128	61	33	-23	72.39475119
Reljic et al.(2021)a	%body fat	ES	42.9	7.5	41.1	7.8	32	-1.8	7.654410493
		CON	45.2	7	44.4	7.7	33	-0.8	7.374957627
	SBP	ES	144	16	133	11	36	-11	14.17744688
		CON	136	15	136	12	29	0	13.74772708
	DBP	ES	94	11	86	7	29	-8	9.643650761
		CON	86	11	86	9	17	0	10.14889157
	WC	ES	117.2	20	117.2	17.9	32	0	19.0370691
		CON	110.3	12.3	109.3	13.3	33	-1	12.82926342
	body mass	ES	116.7	28.1	112.7	27.6	32	-4	27.85336604
		CON	106.4	20.2	103.6	20.9	33	-2.8	20.55893966
	VO2max	ES	20.5	5.1	23.5	6	29	3	5.604462508
		CON	17.1	4.8	16.9	5.5	17	-0.2	5.185556865
	HDL	ES	1.2	0.3	1.3	0.3	29	0.1	0.3
Reljic et al.(2021)b		CON	1.4	0.3	1.3	0.3	17	-0.1	0.3
	LDL	ES	3.9	0.7	3.8	0.7	29	-0.1	0.7
		CON	3.9	0.8	3.6	0.7	17	-0.3	0.754983444
	triglycerides	ES	1.6	0.6	1.6	0.6	29	0	0.6

		CON	1.7	0.9	1.5	0.7	17	-0.2	0.818535277
	%body fat	ES	44.3	7.1	42.9	8.2	29	-1.4	7.709085549
		CON	48.1	5.9	47.5	6.3	17	-0.6	6.109828148
	SBP	ES	141	14	130	13	29	-11	13.52774926
		CON	134	16	134	12	17	0	14.4222051
	DBP	ES	91	12	84	9	32	-7	10.81665383
		CON	85	14	85	10	33	0	12.489996
	WC	ES	123.5	18.2	117.1	16.9	29	-6.4	17.58607404
		CON	112.6	13.6	112.6	14.8	17	0	14.23797738
	body mass	ES	126.5	27.9	122.3	27.8	29	-4.2	27.85013465
		CON	106.3	19.1	103.7	19.8	17	-2.6	19.45944501
	VO2max	ES	22.6	5.5	25.6	5.6	26	3	5.550675635
		CON	20.6	7.4	20.2	7.9	26	-0.4	7.662245102
	HDL	ES	49	10	48	11	26	-1	10.53565375
		CON	56	12	54	12	26	-2	12
	LDL	ES	144	27	143	27	26	-1	27
		CON	154	33	147	26	26	-7	30.11644069
Reljic et al.(2022)a	triglycerides	ES	132	56	128	44	26	-4	51.06858134
		CON	148	73	130	64	26	-18	68.94200461
	body mass	ES	117	26.1	113	25.2	26	-4	25.66183937
		CON	104.4	20.5	101.5	21.5	26	-2.9	21.01784956
	%body fat	ES	42.3	7.6	40.7	8.1	26	-1.6	7.861933604
		CON	46.1	7	44.9	7.5	26	-1.2	7.262919523
	SBP	ES	144	17	133	11	26	-11	14.93318452
		CON	138	13	137	11	26	-1	12.12435565
	DBP	ES	94	11	86	8	26	-8	9.848857802

		CON	89	9	87	7	26	-2	8.185352772
	WC	ES	116	19	110	18	26	-6	18.52025918
		CON	109	11	107	11	26	-2	11
	TC	ES	214	33	213	35	26	-1	34.04408906
		CON	235	42	222	33	22	-13	38.301436
	VO2max	ES	35	5.7	35	6.1	19	0	5.910160742
		CON	37.5	8.2	31.6	8.2	26	-5.9	8.2
	HDL	ES	54	11	57	11	19	3	11
		CON	62	16	62	18	26	0	17.08800749
	LDL	ES	147	29	149	33	19	2	31.19294792
		CON	139	33	146	34	26	7	33.51119216
	triglycerides	ES	120	68	120	91	19	0	81.95730596
		CON	108	68	119	71	26	11	69.5485442
	body mass	ES	87.5	18.8	86.3	18.4	19	-1.2	18.60322553
		CON	83.7	20.2	83.4	19.2	26	-0.3	19.71902635
Reljic et al.(2022)b	%body fat	ES	29.9	7.7	29.1	7.9	19	-0.8	7.80192284
		CON	29.6	9.9	29.5	9.1	26	-0.1	9.525229656
	SBP	ES	135	12	126	12	19	-9	12
		CON	126	12	127	11	26	1	11.53256259
	DBP	ES	89	8	84	8	19	-5	8
		CON	84	6	86	7	26	2	6.557438524
	WC	ES	96.8	14.1	95.3	12.9	19	-1.5	13.53994092
		CON	89.8	13.7	89.7	13.5	26	-0.1	13.6011029
	TC	ES	226	36	228	39	19	2	37.58989226
		CON	222	45	228	45	26	6	45
Reljic et al.(2023)	VO2max	ES	22.6	5.5	25.6	5.6	20	3	5.550675635

		CON	20.6	7.4	20.2	7.9	18	-0.4	7.662245102
	HDL	ES	49.2	10	49.4	11.2	20	0.2	10.65082156
		CON	55.1	12.3	53	12.1	18	-2.1	12.20122945
	LDL	ES	147.8	28.8	143.9	26.8	20	-3.9	27.85390457
		CON	148.7	29.9	148.2	22.6	18	-0.5	27.00055555
	triglycerides	ES	132	56	128	44	20	-4	51.06858134
		CON	148	73	130	64	18	-18	68.94200461
	body mass	ES	117	26.1	113	25.2	20	-4	25.66183937
		CON	104.4	20.5	101.5	21.5	18	-2.9	21.01784956
	%body fat	ES	42.3	7.6	40.7	8.1	20	-1.6	7.861933604
		CON	46.1	7	44.9	7.5	18	-1.2	7.262919523
	SBP	ES	144	17	133	11	20	-11	14.93318452
		CON	138	13	137	11	18	-1	12.12435565
	DBP	ES	94	11	86	8	20	-8	9.848857802
		CON	89	9	87	7	18	-2	8.185352772
	WC	ES	116.8	21.3	110.2	18.1	20	-6.6	19.89396894
		CON	113.3	13.4	111.8	15.4	18	-1.5	14.50379261
	TC	ES	219.3	35.1	215.3	36.2	20	-4	35.66272564
		CON	218	37.2	217	31.1	18	-1	34.55618613
	body mass	ES	76.4	16.2	76.2	16.8	12	-0.2	16.50817979
		CON	82.8	19.1	83.1	18.9	6	0.3	19.00078946
Schubert et al.(2017)	%body fat	ES	25.7	8.4	23.8	8.1	12	-1.9	8.254089895
		CON	20.8	7.8	21	8	6	0.2	7.901898506
	WC	ES	84.4	11.1	85.1	10.6	12	0.7	10.85863712
		CON	79	7	79.5	7.4	6	0.5	7.208328516
Scoubeau et al.(2023)	VO2max	ES	30.9	4.5	36.3	5.3	14	5.4	4.948737213

		CON	28.5	8.5	28.4	6.9	14	-0.1	7.823681998
	body mass	ES	18.4	5.8	18.4	5.9	17	0	5.850640991
		CON	22	8.3	23	8.5	14	1	8.401785525
	%body fat	ES	31.6	5.8	31.5	5.9	17	-0.1	5.850640991
		CON	31.3	8.3	31.4	8.5	14	0.1	8.401785525
Stojanović et al.(2021)	HDL	ES	1.31	0.34	1.37	0.38	86	0.06	0.361662826
		CON	1.5	0.37	1.47	0.4	82	-0.03	0.385875628
	LDL	ES	3.9	1.22	3.52	1.04	86	-0.38	1.140701539
		CON	3.84	1.22	3.84	1.15	82	0	1.18654962
	fasting glucose	ES	5.96	1.76	5.64	1.68	86	-0.32	1.721394783
		CON	6.3	2.24	6.44	2.28	82	0.14	2.260265471
	triglycerides	ES	1.66	0.74	1.65	0.66	86	-0.01	0.703420216
		CON	1.68	0.62	1.71	0.65	82	0.03	0.635531274
Venegas-Carro et al.(2023)	VO2max	ES	42.2	6.4	45.7	6.5	16	3.5	6.450581369
		CON	40.6	6.8	41.3	7	15	0.7	6.902173571
Wanders et al.(2021)	HDL	ES	1.36	0.39	1.33	0.4	17	-0.03	0.395094925
		CON	1.4	0.38	1.32	0.39	18	-0.08	0.38509739
	LDL	ES	3.13	0.6	2.96	0.57	17	-0.17	0.585576639
		CON	3.13	0.58	3.11	0.6	18	-0.02	0.590254183
	triglycerides	ES	1.49	0.67	2.04	0.84	19	0.55	0.769220385
		CON	1.41	0.62	2	0.78	18	0.59	0.713582511
	TC	ES	5.15	0.66	5.15	0.6	19	0	0.632139225
		CON	5.24	0.77	5.47	0.81	18	0.23	0.790759129
Wong et al.(2024)	VO2max	ES	36.6	6.3	37.4	6.5	11	0.8	6.402343321
		CON	36.1	6.2	36.1	6.1	8	0	6.150609726
	fasting glucose	ES	82.3	7.6	79.5	5.4	11	-2.8	6.773477689

		CON	79.5	2.7	78.5	6.4	8	-1	5.565069631
	triglycerides	ES	59.5	35.4	61	22.7	11	1.5	31.06235664
		CON	71.8	25.5	60.4	28.3	8	-11.4	27.00907255
	body mass	ES	60.5	7.4	60.2	7.8	11	-0.3	7.607890641
		CON	57.9	7.7	58.1	7.5	8	0.2	7.601973428
	%body fat	ES	22.8	6.5	23.6	5.9	11	0.8	6.221736092
		CON	19.5	5.3	19.3	5.6	8	-0.2	5.456189146
	SBP	ES	105	12	106	9	11	1	10.81665383
		CON	113	8	108	10	8	-5	9.16515139
	DBP	ES	63	9	65	4	11	2	7.810249676
		CON	68	7	66	11	8	-2	9.643650761
Yin et al.(2024)	VO2max	ES	NA	NA	NA	NA	14	3.3	3.2
		CON	NA	NA	NA	NA	15	-2.9	3.5
Zhou et al.(2025)	VO2max	ES	33.14	4.97	37.61	3.87	14	4.47	4.521493116
		CON	31.67	5.09	32.06	4.95	13	0.39	5.02146393
	body mass	ES	76.81	14.76	74.29	14.55	14	-2.52	14.65612841
		CON	75.07	11.5	75.34	11.91	13	0.27	11.71038428
	%body fat	ES	41.71	4.71	39.59	4.35	14	-2.12	4.540715803
		CON	41.97	4.82	42.17	4.95	13	0.2	4.886297167

Table S4. Risk of bias within studies

Author, year	Selection bias	Study design	Confounders	Blinding	Data collection method	Withdrawals and dropouts	Intervention integrity measured (Q2)	Analyses Intention to treat	Global rating
Banitaleb et al.(2019)	moderate	strong	strong	moderate	strong	strong	yes	NR	strong
Bhammar et al.(2017)	weak	strong	strong	weak	strong	moderate	yes	NR	weak
Boer et al.(2014)	weak	strong	weak	weak	strong	weak	no	NR	weak
Boreham et al.(2000)	moderate	strong	weak	weak	strong	strong	no	NR	weak
Boreham et al.(2005)	weak	strong	strong	moderate	strong	strong	yes	yes	moderate
Chin et al.(2020)	weak	strong	weak	weak	strong	moderate		NR	weak
Dogra et al.(2019)	moderate	strong	strong	moderate	strong	strong	no	NR	strong
Elley et al.(2006)	moderate	strong	strong	weak	strong	moderate	no	NR	moderate
Gillen et al.(2016)	moderate	strong	weak	weak	strong	weak	no	NR	weak
Jabbour et al.(2017)	weak	strong	weak	weak	strong	strong	no	NR	weak
Metcalfe et al.(2011)	weak	strong	strong	weak	strong	weak	yes	NR	weak
Michael et al.(2021)	weak	strong	weak	moderate	strong	strong	no	NR	weak
Perkin et al.(2019)	moderate	strong	strong	weak	strong	moderate	yes	yes	moderate
Reljic et al.(2020)	moderate	strong	weak	moderate	strong	strong	no	NR	moderate
Reljic et al.(2021)a	moderate	strong	strong	weak	strong	strong	yes	yes	moderate
Reljic et al.(2021)b	weak	strong	weak	moderate	strong	weak	no	NR	weak
Reljic et al.(2022)a	moderate	strong	strong	moderate	strong	strong	no	NR	strong
Reljic et al.(2022)b	weak	strong	weak	weak	strong	strong	no	NR	weak
Reljic et al.(2023)	weak	strong	strong	moderate	strong	moderate	yes	NR	moderate
Schubert et al.(2017)	moderate	strong	weak	moderate	strong	moderate	yes	NR	moderate

Scoubeau et al.(2023)	moderate	strong	strong	moderate	strong	strong	yes	yes	strong
Stojanović et al.(2021)	weak	strong	strong	moderate	strong	weak	no	NR	weak
Venegas-Carro et al.(2023)	weak	strong	strong	weak	strong	strong	no	NR	weak
Wanders et al.(2021)	moderate	strong	weak	weak	strong	strong	yes	NR	moderate
Wong et al.(2024)	moderate	strong	strong	weak	strong	strong	no	NR	moderate
Yin et al.(2024)	moderate	strong	strong	weak	strong	moderate	no	NR	moderate
Zhou et al.(2025)	weak	strong	strong	moderate	strong	moderate	yes	yes	moderate

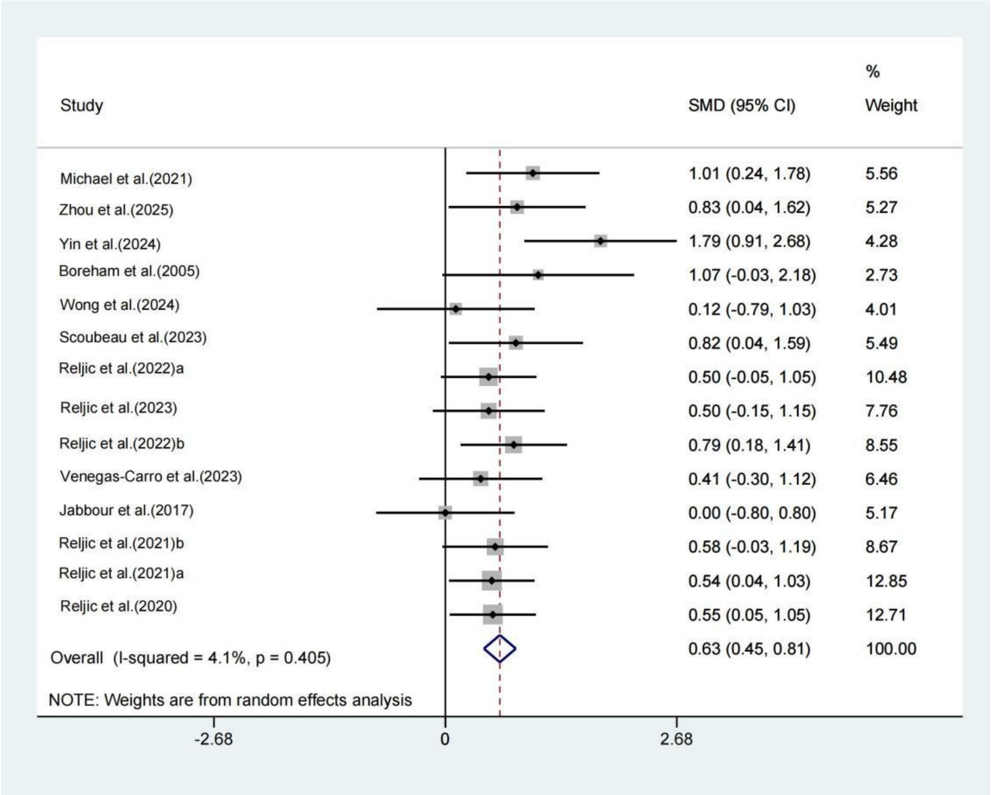

Figure S1. The forest plot showing the effects sizes (SMD) of ES interventions on VO_2max

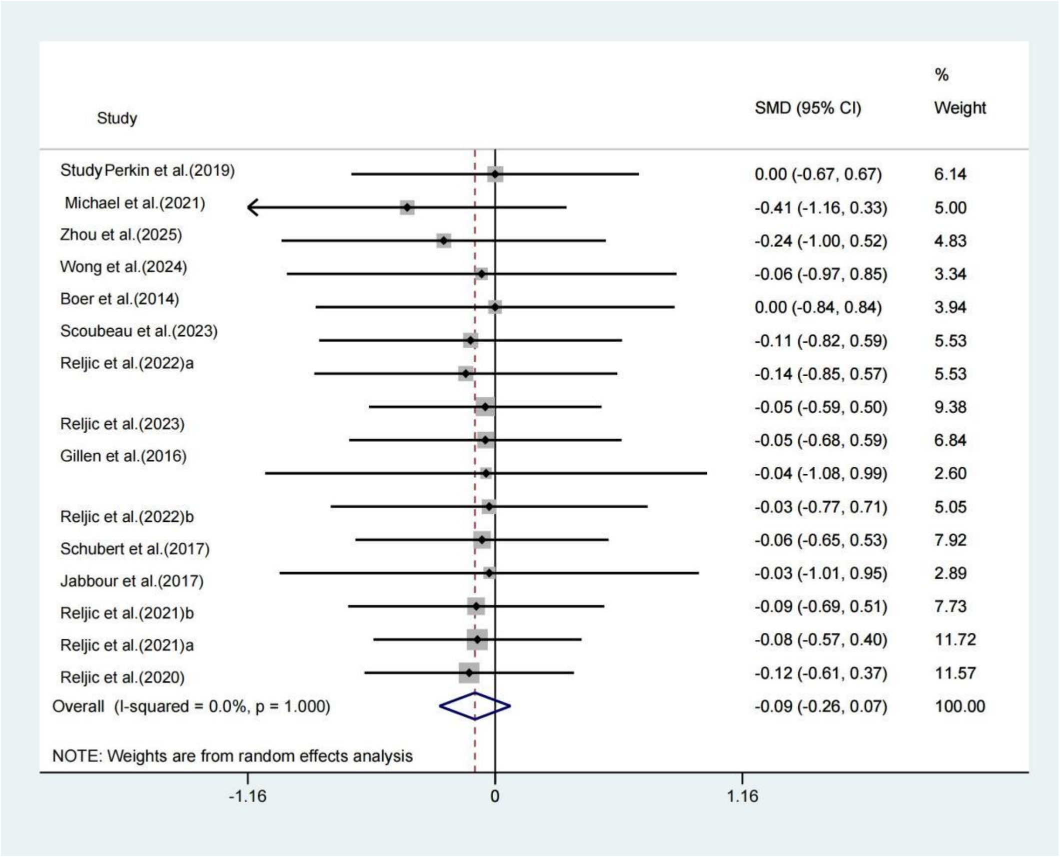

Figure S2. The forest plot showing the effects sizes (SMD) of ES interventions on body mass

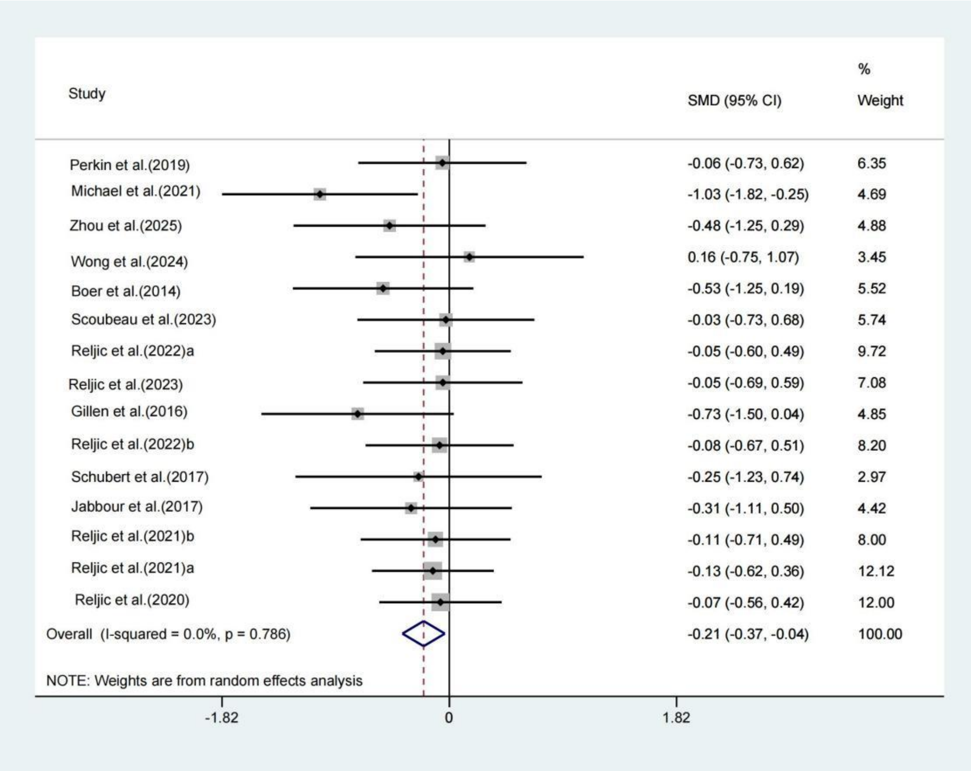

Figure S3. The forest plot showing the effects sizes (SMD) of ES interventions on BF%

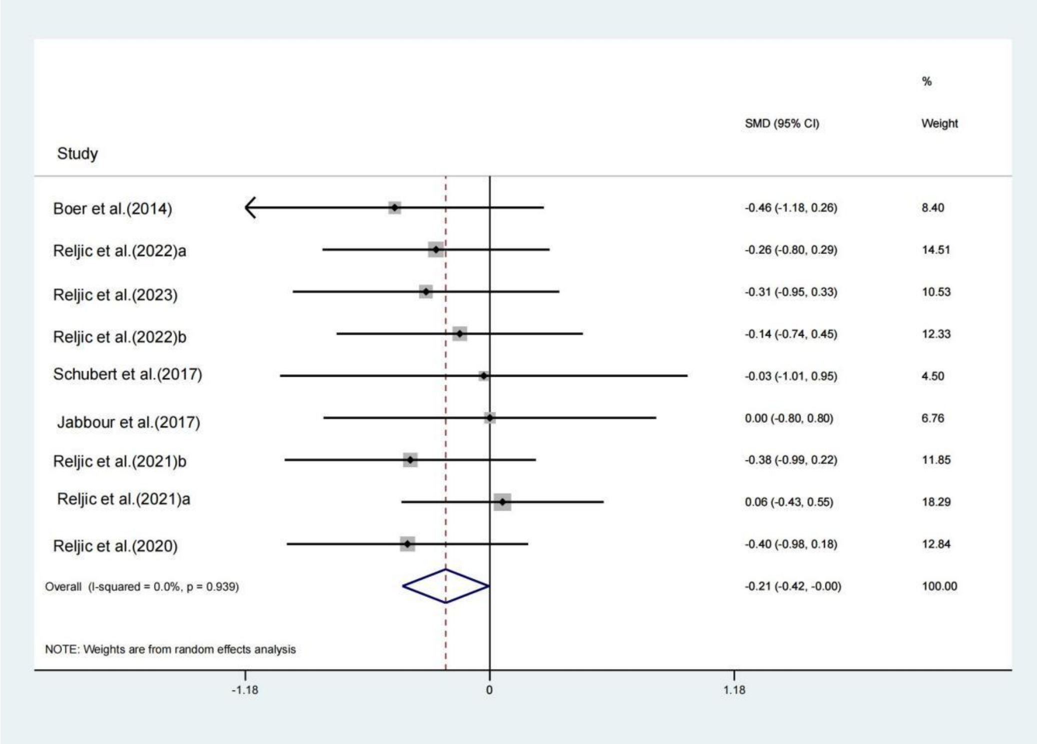

Figure S4. The forest plot showing the effects sizes (SMD) of ES interventions on WC

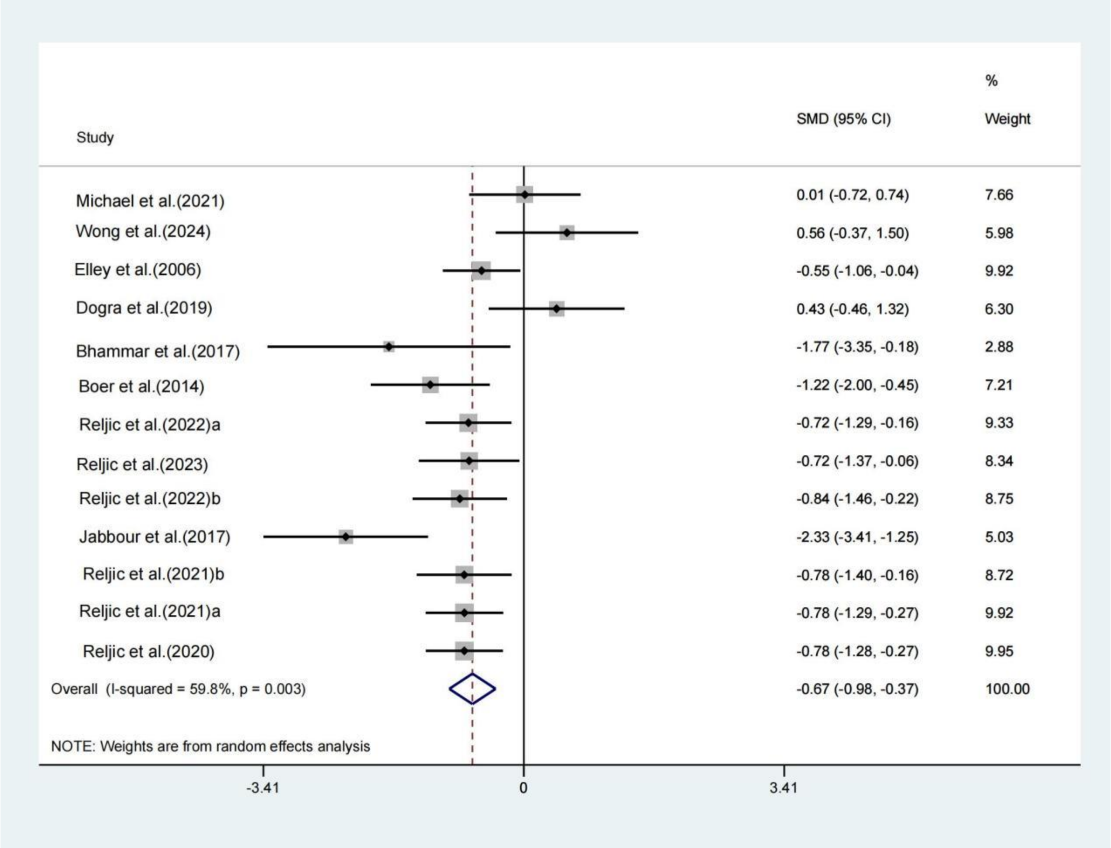

Figure S5. The forest plot showing the effects sizes (SMD) of ES interventions on SBP

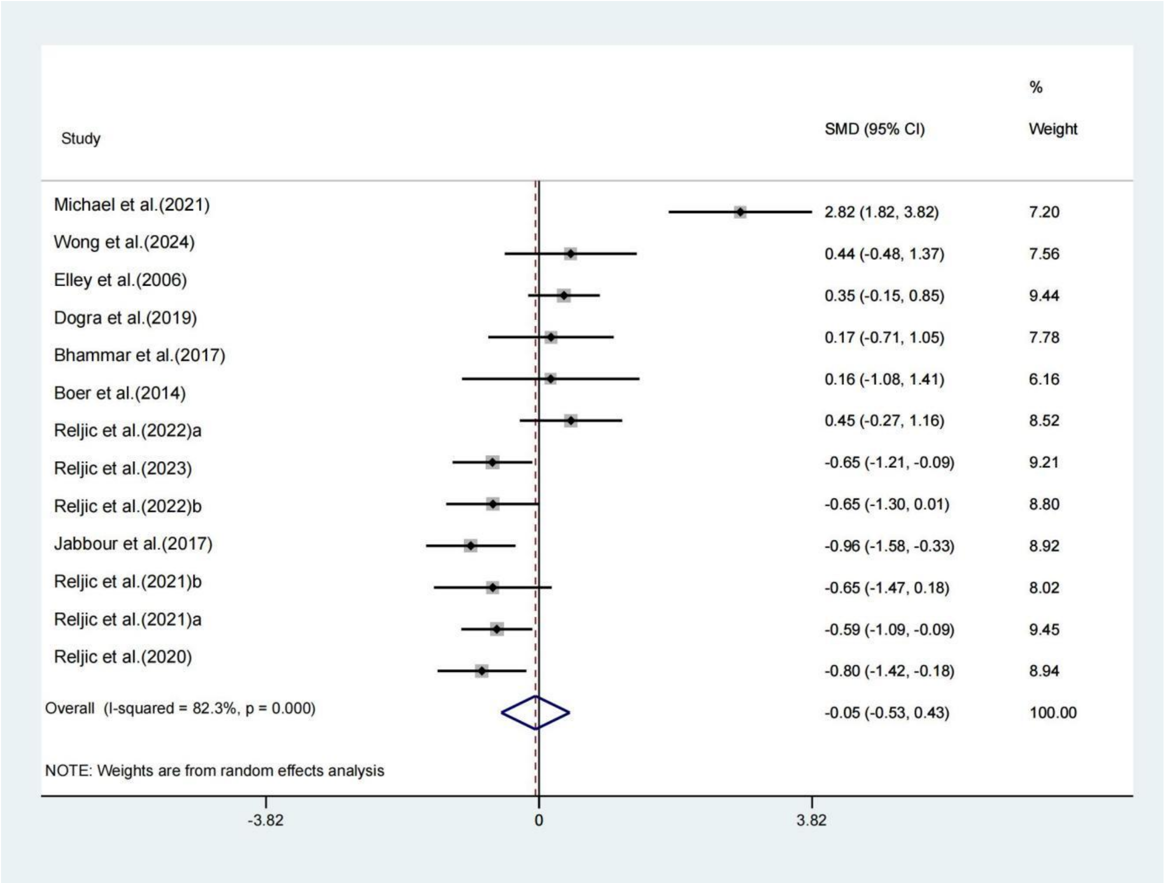

Figure S6. The forest plot showing the effects sizes (SMD) of ES interventions on DBP

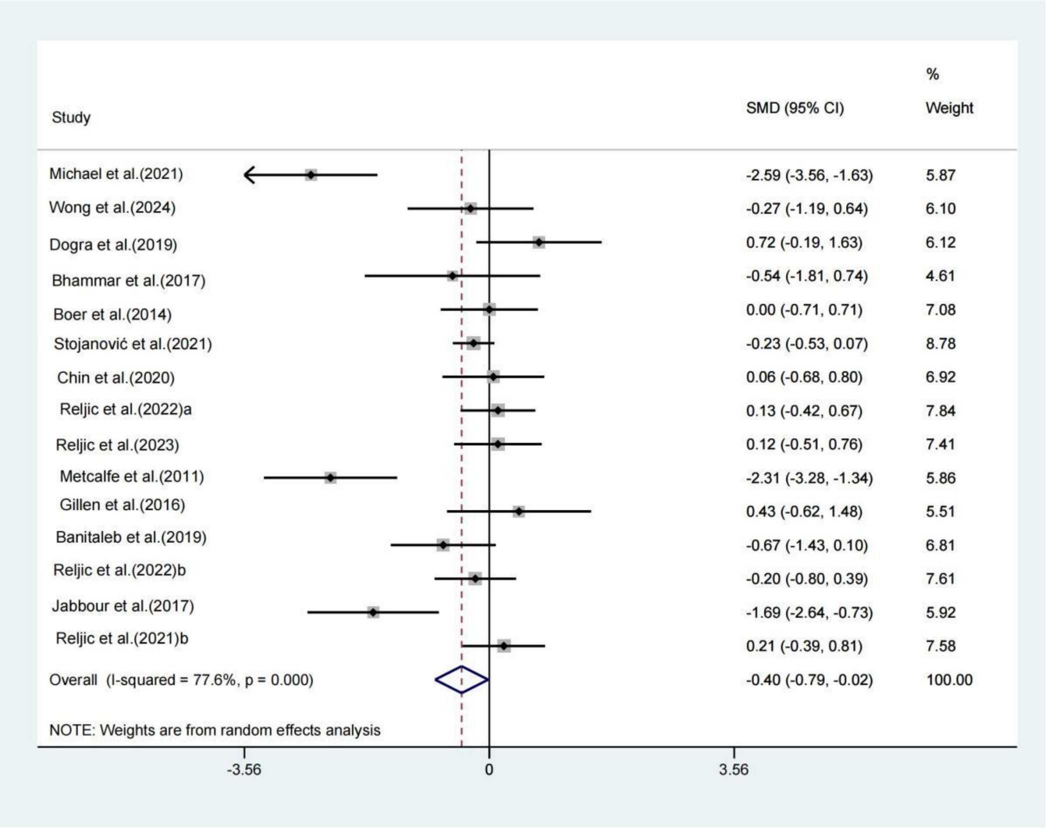

Figure S7. The forest plot showing the effects sizes (SMD) of ES interventions on FBG

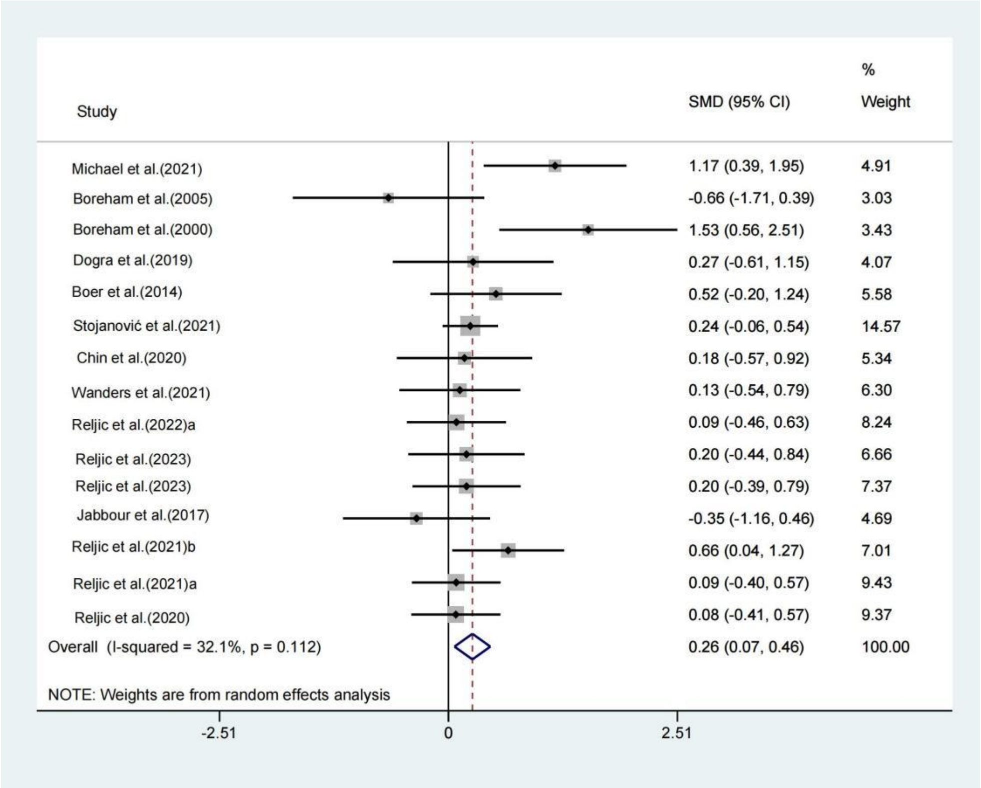

Figure S8. The forest plot showing the effects sizes (SMD) of ES interventions on HDL

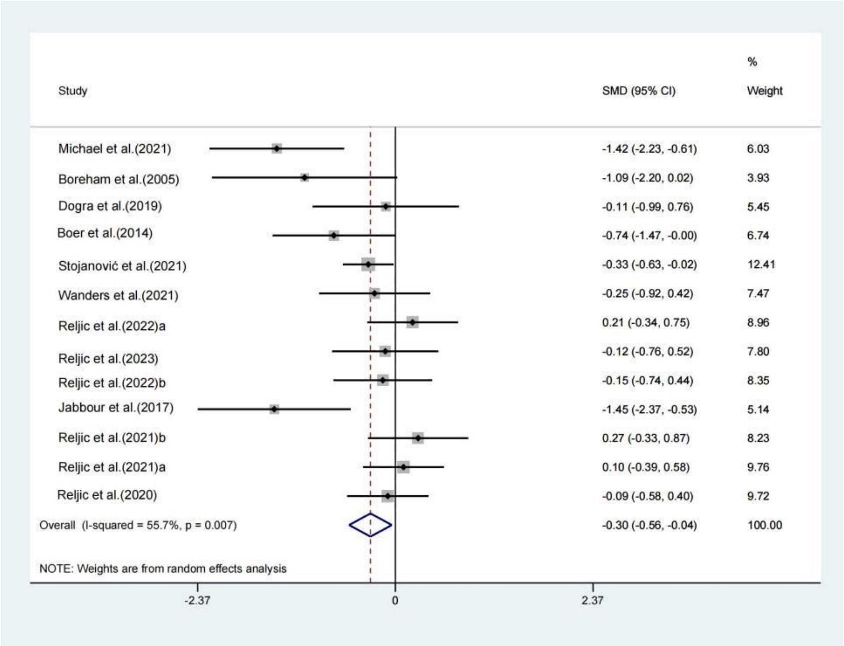

Figure S9. The forest plot showing the effects sizes (SMD) of ES interventions on LDL

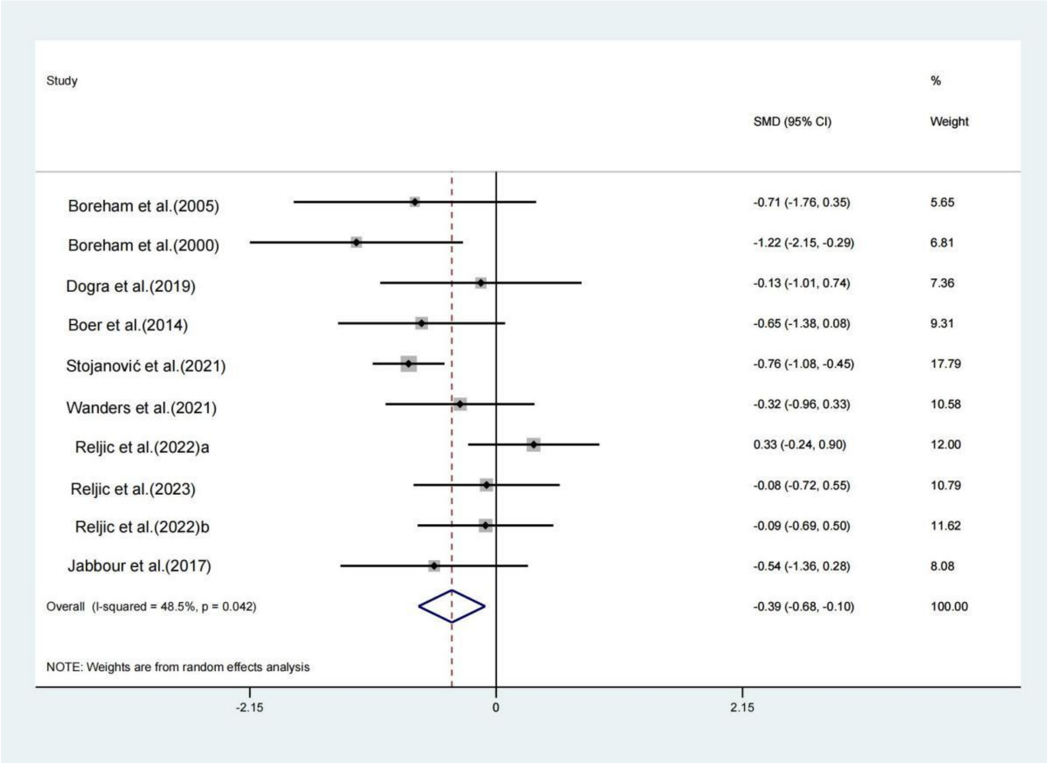

Figure S10. The forest plot showing the effects sizes (Hedge' s g) of ES interventions on TC

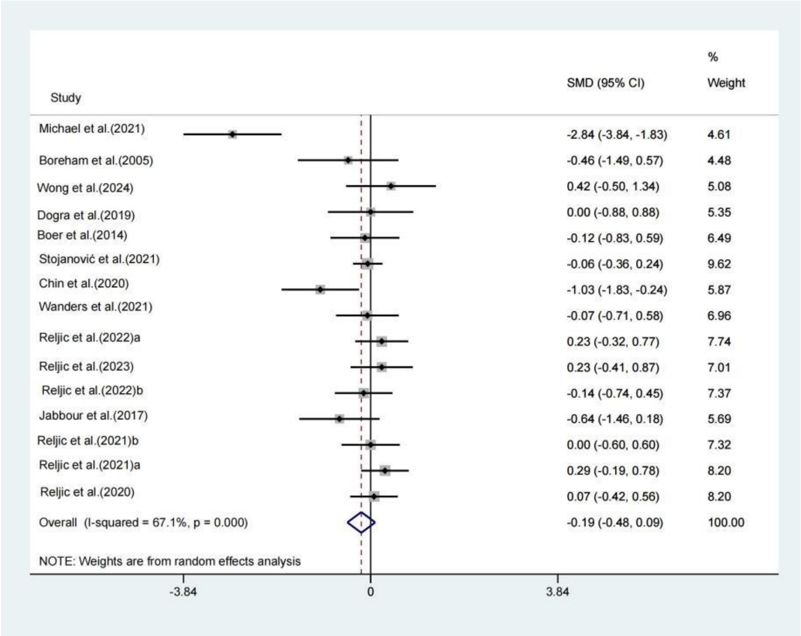

Figure S11. The forest plot showing the effects sizes (SMD) of ES interventions on TG

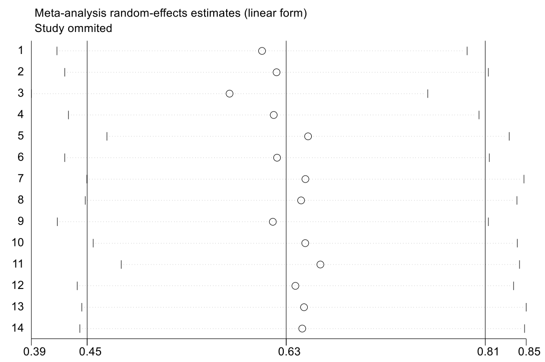

Figure S12. The forest plot showing the sensitivity analysis of ES interventions on VO_2max for each study

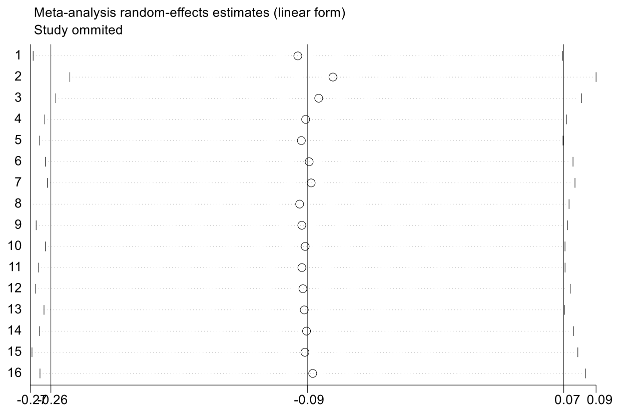

Figure S13. The forest plot showing the sensitivity analysis of ES interventions on body mass for each study

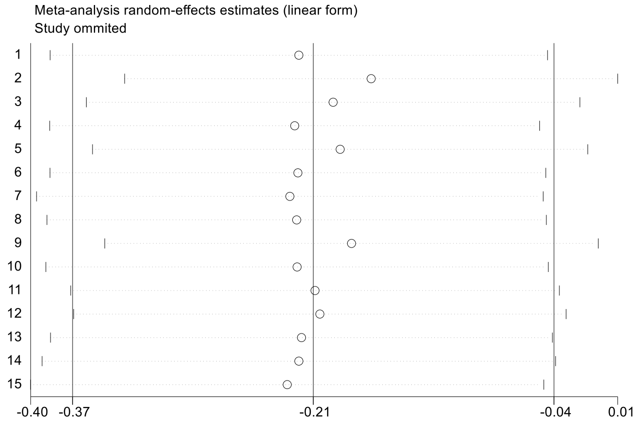

Figure S14. The forest plot showing the sensitivity analysis of ES interventions on BF% for each study

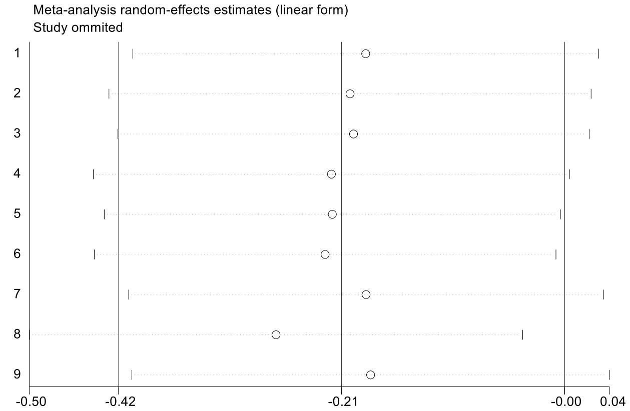

Figure S15. The forest plot showing the sensitivity analysis of ES interventions on WC for each study

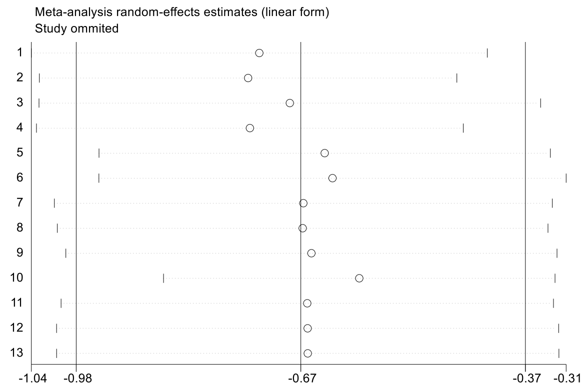

Figure S16. The forest plot showing the sensitivity analysis of ES interventions on SBP for each study

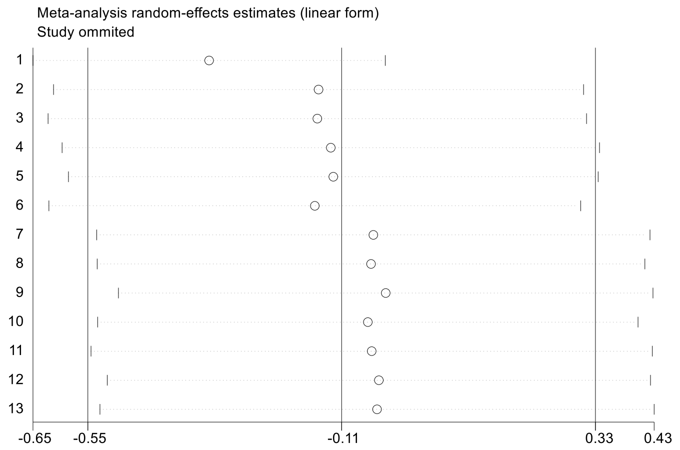

Figure S17. The forest plot showing the sensitivity analysis of ES interventions on DBP for each study

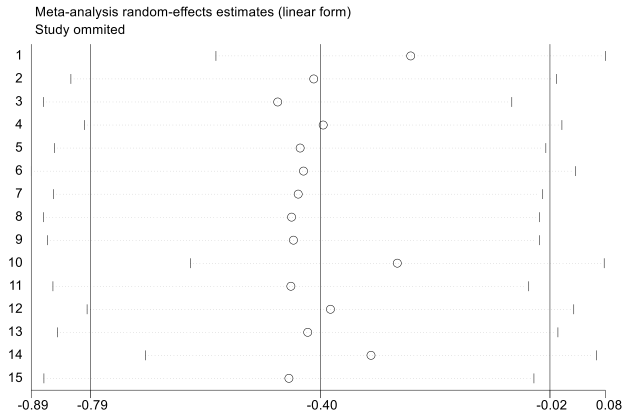

Figure S18. The forest plot showing the sensitivity analysis of ES interventions on FBG for each study

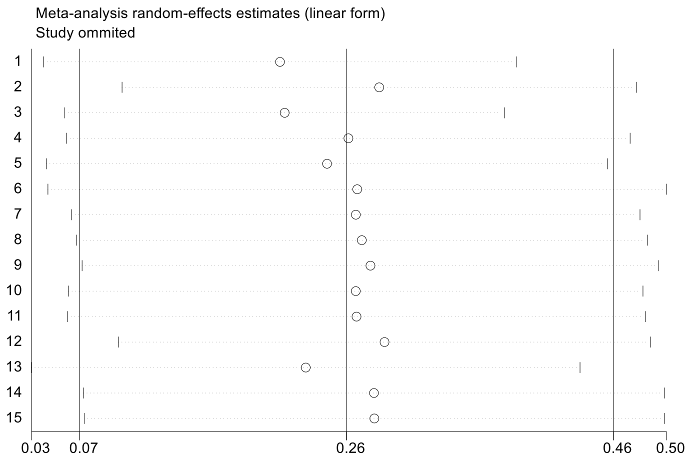

Figure S19. The forest plot showing the sensitivity analysis of ES interventions on HDL for each study

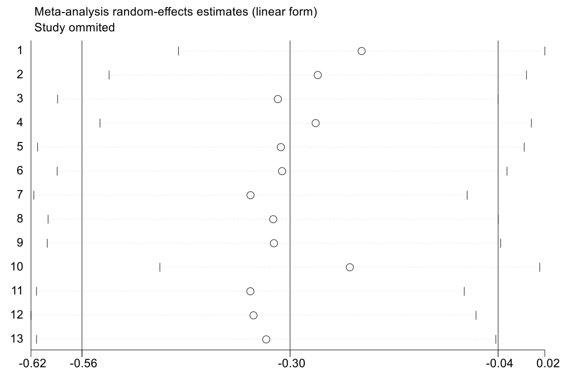

Figure S20. The forest plot showing the sensitivity analysis of ES interventions on LDL for each study

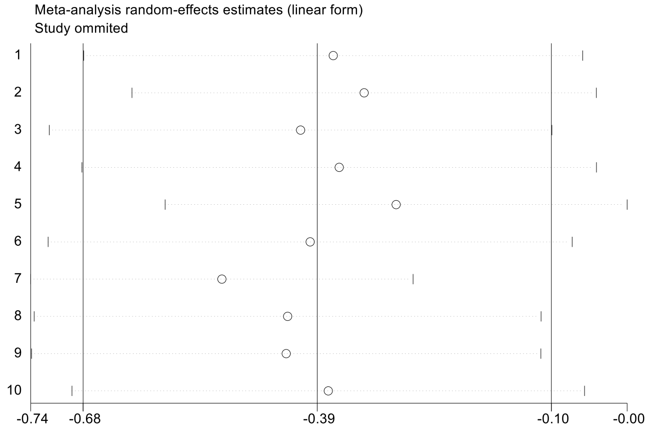

Figure S21. The forest plot showing the sensitivity analysis of ES interventions on TC for each study

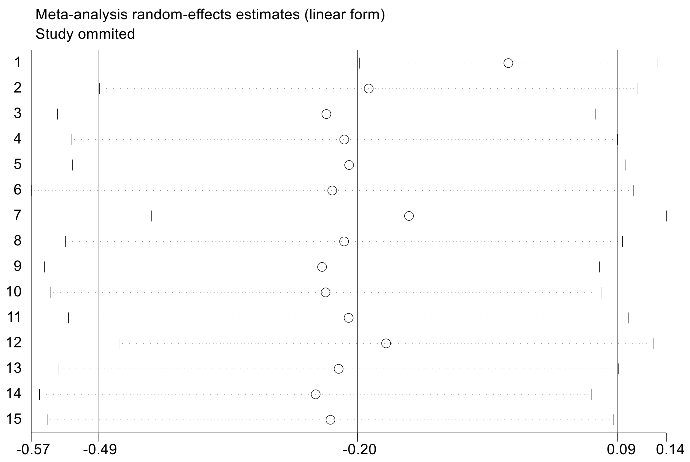

Figure S22. The forest plot showing the sensitivity analysis of ES interventions on TG for each study
